# Transgenic Suppression of *AGAMOUS* Genes in Apple Reduces Fertility and Increases Floral Attractiveness

**DOI:** 10.1371/journal.pone.0159421

**Published:** 2016-08-08

**Authors:** Amy L. Klocko, Ewa Borejsza-Wysocka, Amy M. Brunner, Olga Shevchenko, Herb Aldwinckle, Steven H. Strauss

**Affiliations:** 1 Department of Forest Ecosystems and Society, Oregon State University, Corvallis, Oregon, United States of America; 2 Section of Plant Pathology and Plant-Microbe Biology, School of Integrative Plant Science, Cornell University, Geneva, New York, United States of America; 3 Department of Forest Resources and Environmental Conservation, Virginia Tech, Blacksburg, Virginia, United States of America; United States Department of Agriculture, UNITED STATES

## Abstract

We investigated the ability of RNA interference (RNAi) directed against two co-orthologs of *AGAMOUS* (*AG*) from *Malus domestica* (domestic apple, *MdAG*) to reduce the risks of invasiveness and provide genetic containment of transgenes, while also promoting the attractiveness of flowers for ornamental usage. Suppression of two *MdAG*-like genes, *MdMADS15* and *MdMADS22*, led to the production of trees with highly showy, polypetalous flowers. These “double-flowers” had strongly reduced expression of both *MdAG-*like genes. Members of the two other clades within in the *MdAG* subfamily showed mild to moderate differences in gene expression, or were unchanged, with the level of suppression approximately proportional to the level of sequence identity between the gene analyzed and the RNAi fragment. The double-flowers also exhibited reduced male and female fertility, had few viable pollen grains, a decreased number of stigmas, and produced few viable seeds after cross-pollination. Despite these floral alterations, RNAi-*AG* trees with double-flowers set full-sized fruit. Suppression or mutation of apple *AG*-like genes appears to be a promising method for combining genetic containment with improved floral attractiveness.

## Introduction

Many ornamental and fruit tree species are grown outside of their native range as exotics, and thus have the potential to become invasive. In addition, some species are starting to be cultivated as specialized genetically engineered (GE) varieties, which could potentially interbreed with wild, feral, or traditionally grown relatives (e.g., Arctic Apple [1]). When needed, sterile trees would have the benefits of reduced invasiveness, decreased production of allergenic pollen, and mitigation of unwanted gene flow [2].

There are several methods which can be used to obtain sterile trees, including traditional approaches such as triploid breeding, random mutagenesis, and the creation of wide hybrids with disturbed meiosis [3]. However, the very long juvenile period of many trees, and the need to rebreed each variety in conjunction with these extensive genomic modifications, make these methods extremely time-consuming. Existing ablation-based GE methods have led to the successful creation of male-sterile pines, eucalypts, and poplars [4, 5]. With the exception of recent findings in poplar [6] there is a current dearth of GE-based female-sterile or bisexually-sterile trees. Some popular ornamental varieties of trees, such as Bradford pear, are both bisexual and invasive [7]. GE technology could be used to create bisexually sterile versions of such trees, greatly reducing their potential for spread via sexual propagules, while keeping their basic vegetative traits and adaptations intact.

The *AGAMOUS* (*AG*) gene is an attractive target for achieving complete floral sterility in trees. Studies in model species have demonstrated that AG acts early in floral organ determination to specify anther and carpel identity, and also to regulate floral meristem determinacy. The loss of *AG* results in the conversion of reproductive organs, anthers and carpels, to non-reproductive organs, sepals and petals; which, combined with reduction of floral meristem determinacy, leads to a distinctive flower-in-flower phenotype [8–10]. However, there is little information regarding AG function in woody species. Initial characterization of *MdMADS15*, a close homolog of *AG* from apple, showed that this gene is expressed in anthers and carpels, as would be expected for a C-class floral gene [11]. Whether or not *MdMADS15* fulfilled AG function in apple had yet to be determined. Here we present functional characterization data showing that RNA interference (RNAi) against two co-orthologs of *AG* from *Malus domestica* (domestic apple, variety *Galaxy*, a cultivar of Gala) led to striking reductions in both male and female fertility, while still allowing fruits to develop. In addition, the conversion of anthers to petals in RNAi-*AG* trees produced flowers with a highly attractive double-flower phenotype that should enhance the value of such trees as an ornamental variety.

## Results

### Floral phenotype

**Fig 1:**
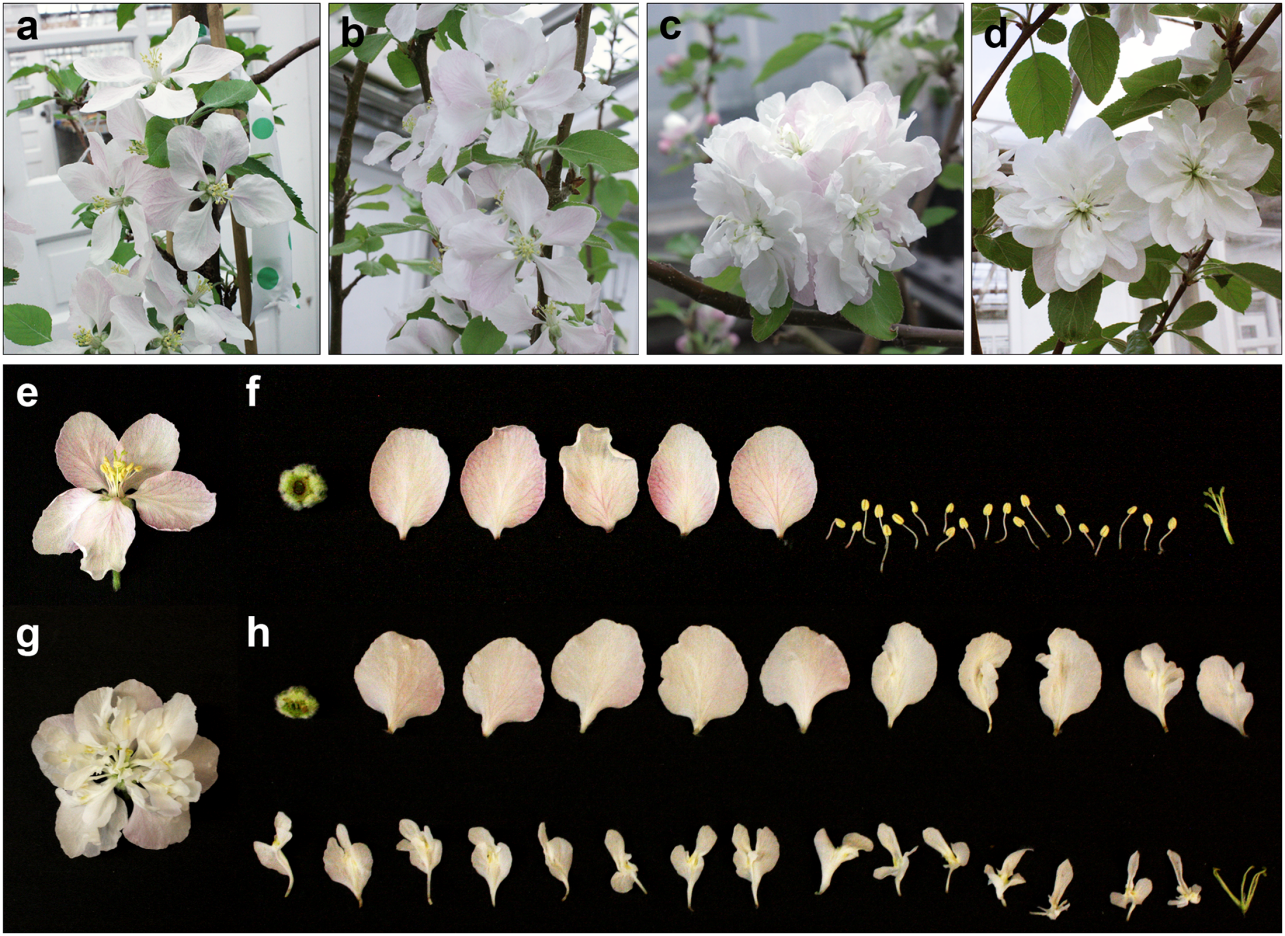
RNAi of apple *AG*-like genes led to showy, polypetalous flowers with anthers converted to petals. (a) Control trees developed flowers with a single whorl of five petals (b) as did four RNAi-*AG* events. (c, d) Four other RNAi-*AG* events developed flowers with additional whorls of petals and a lack of obvious anthers. Dissection of (e, f) non-transgenic control flowers with 5 sepals, 5 petals and 20 anthers; and (g, h) double-flowers with 5 sepals, 5 outer petals, and 20 inner floral organs with both petal and anther characteristics.

The apple genome contains numerous genes predicted to contain MADS-box motifs [12, 13]. Of these, *MdMADS15* and *MdMADS22* are highly similar to *AG* genes from several species (S1 Fig). *MdMADS15* is known to be expressed in apple flower reproductive whorls, but whether this gene, or the closely related *MdMADS22* fulfilled AG function in apple had yet to be tested [11]. We designed an RNAi construct to target both of these *AG-*like genes in domestic apple and obtained a total of eight independent transformation events (S2 Fig, S1 Table). After vegetative propagation, each event was represented by 1–16 ramets (trees). Of the eight total events, four (50%) developed flowers with extra petals (double-flowers); the remaining events had flowers phenotypically similar to those of non-transgenic control trees (single flowers, Fig 1, S1 Table). Phenotypes were consistent within events; all ramets from a given event showed the same floral phenotype (single or double), as did all flowers examined from any given ramet. Survival of the RNAi-*AG* and non-transgenic control trees was identical; all trees used for floral analysis (S1 Table) were alive as of July 2016.

In order to determine the origin of the extra petals, we dissected and imaged control and double-flowers, removing floral organs in order of phylotaxy; starting with the sepals and working inwards. We found that control flowers consisted of five sepals, five petals, 20 anthers, and five stigmas (Fig 1). By comparison, the double-flowers contained five sepals of normal appearance, five outer petals, 20 inner floral organs that exhibited both petal and anther characteristics (petaloid anthers), and a varied number of stigmas. The five outermost petals of the double-flowers were similar in appearance to petals of control flowers, and opened earlier than the 20 inner petaloid anthers (S3 Fig).

### Analysis of male fertility

**Fig 2:**
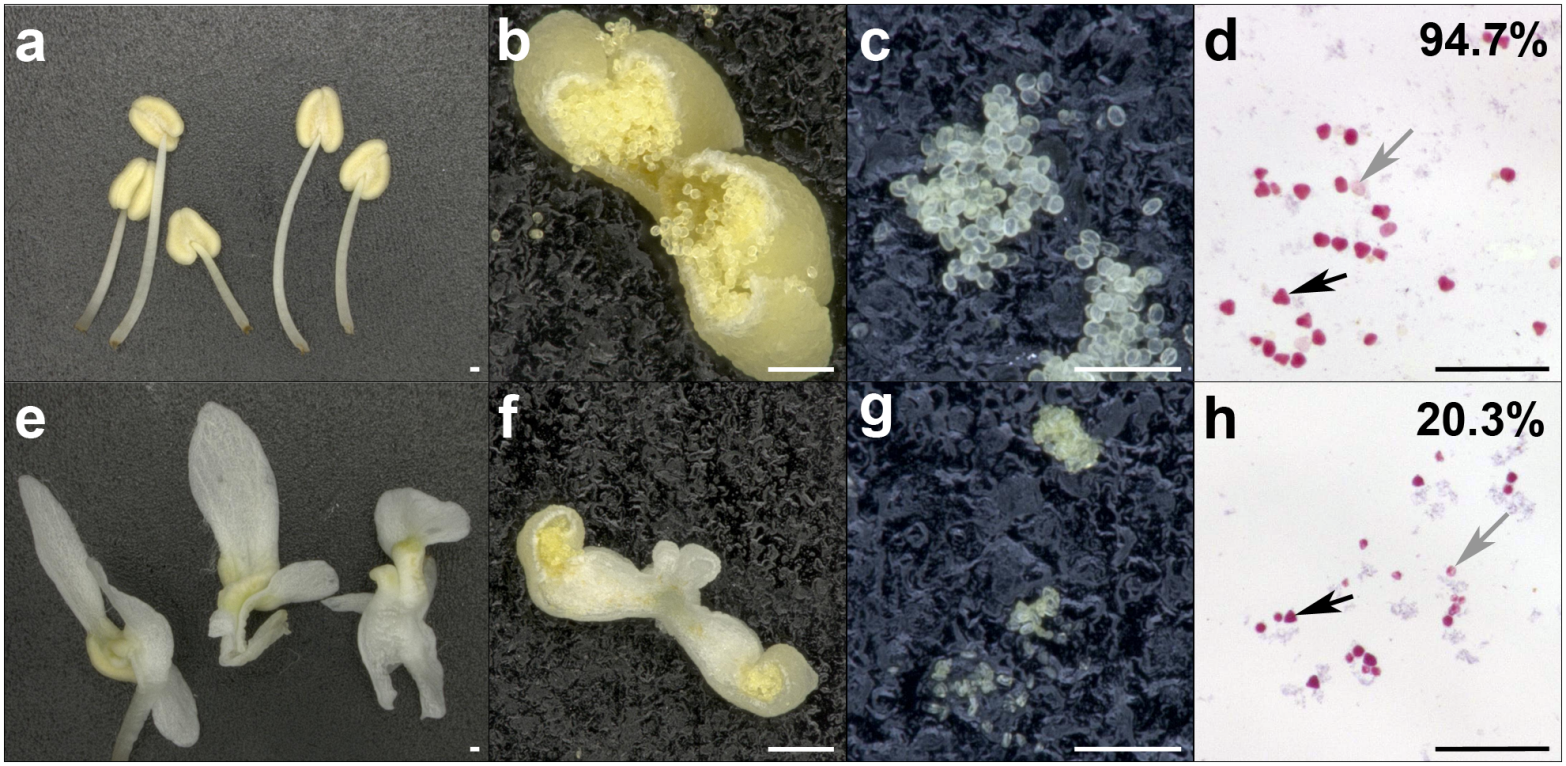
RNAi-*AG* double-flowers contained a small number of viable pollen grains. (a) Whole anthers of control flowers, (b) cross sectioned control anthers with (c) pollen grains which (d) stained viable. Viable grains stained dark pink (black arrow) while non-viable grains remained pale (grey arrow). (e) Whole anthers of double-flowers, (f) cross sectioned petaloid anthers of double-flowers with (g) pollen grains, (h) few of which were viable. The average percent viable pollen grains for control and double-flowers are shown. Bar = 200 μm.

Because the double-flowers had full to partial conversion of anthers to petals, we examined whether these petaloid anthers produced viable pollen grains. These data would help to determine if the flowers retained potential male fertility. Dissection of the innermost petaloid anthers, those with the largest anther-like structures, revealed that they contained some pollen grains (Fig 2). These grains were less numerous and smaller in size than control pollen grains. Alexander staining showed that while 94.7% of the control grains stained dark pink, indicating that these grains were viable, double-flowered events 1600, 1599, 1601 and 1612 had 0.0%, 23.0%, 25.4% and 30.8% viable pollen grains, respectively, all of which were significantly reduced as compared to pollen from control flowers (P < .001). Averaging across double-flowered events showed that pollen viability for these trees was 20.3%. In addition, while control pollen grains were released from anthers, the pollen grains contained within petaloid anthers did not release and thus were obtained by hand sectioning.

### Analysis of female floral structures

**Fig 3:**
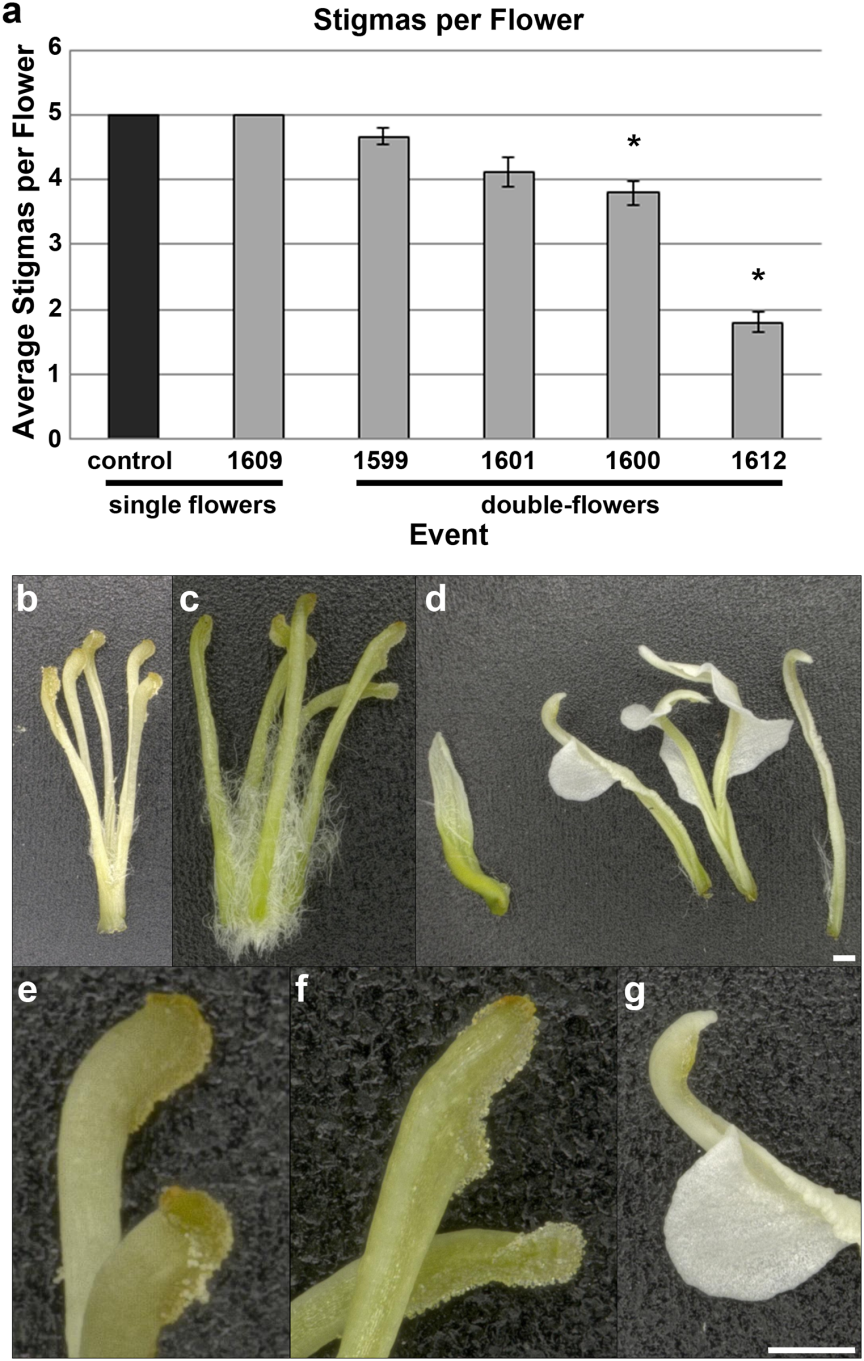
RNAi-*AG* double-flowers had reduced numbers of stigmas and partial conversion of styles to petals. (a) Quantification of the average numbers of stigmas per flower revealed that double-flower events 1600 and 1612 had significantly fewer stigmas than control flowers. Bars show standard error of the mean, asterisks indicate significant differences (P < .01). (b, e) Microscopy of stigmas and styles from control, (c, f) single-flowered transgenic, and (d, g) double-flowered trees showed that stigmas from double-flowers were smaller than those of control flowers and often had projections of petal-like tissue. Panels b-d and e-g were collected at the same respective magnifications, bar = 500 μm.

Inspection of the innermost whorl of the double-flowers showed that they contained a variable number of stigmas, with events 1600 and 1612 having significantly fewer stigmas than control flowers (Fig 3, P < .01). While control and single RNAi-*AG* flowers had well-formed stigmas with a textured appearance attached to uniform styles, double-flowers had small, nearly smooth stigmas, and styles with petal-like projections of tissue.

### Gene expression analysis

We used quantitative real-time PCR (qPCR) to determine if floral phenotypes were related to the levels of target gene expression in floral buds. In addition to quantifying the levels of our primary *AG*-like target genes, *MdMADS22* and *MdMADS15*, we also tested *MdMADS14* and *MdMADS19*, close relatives of *SHATTERPROOF* (*SHP*) and *SEEDSTICK* (*STK*), respectively (S1 Fig). Sequence analysis of these genes from the Galaxy cultivar showed 99.8% identity between the gene fragment used for RNAi and the sequence of *MdMADS22*, 96.2% identity for *MdMADS15*, and just 68.5% and 63.3% identity to *MdMADS14* and *MdMADS19*, respectively (S4–S7 Figs).

**Fig 4:**
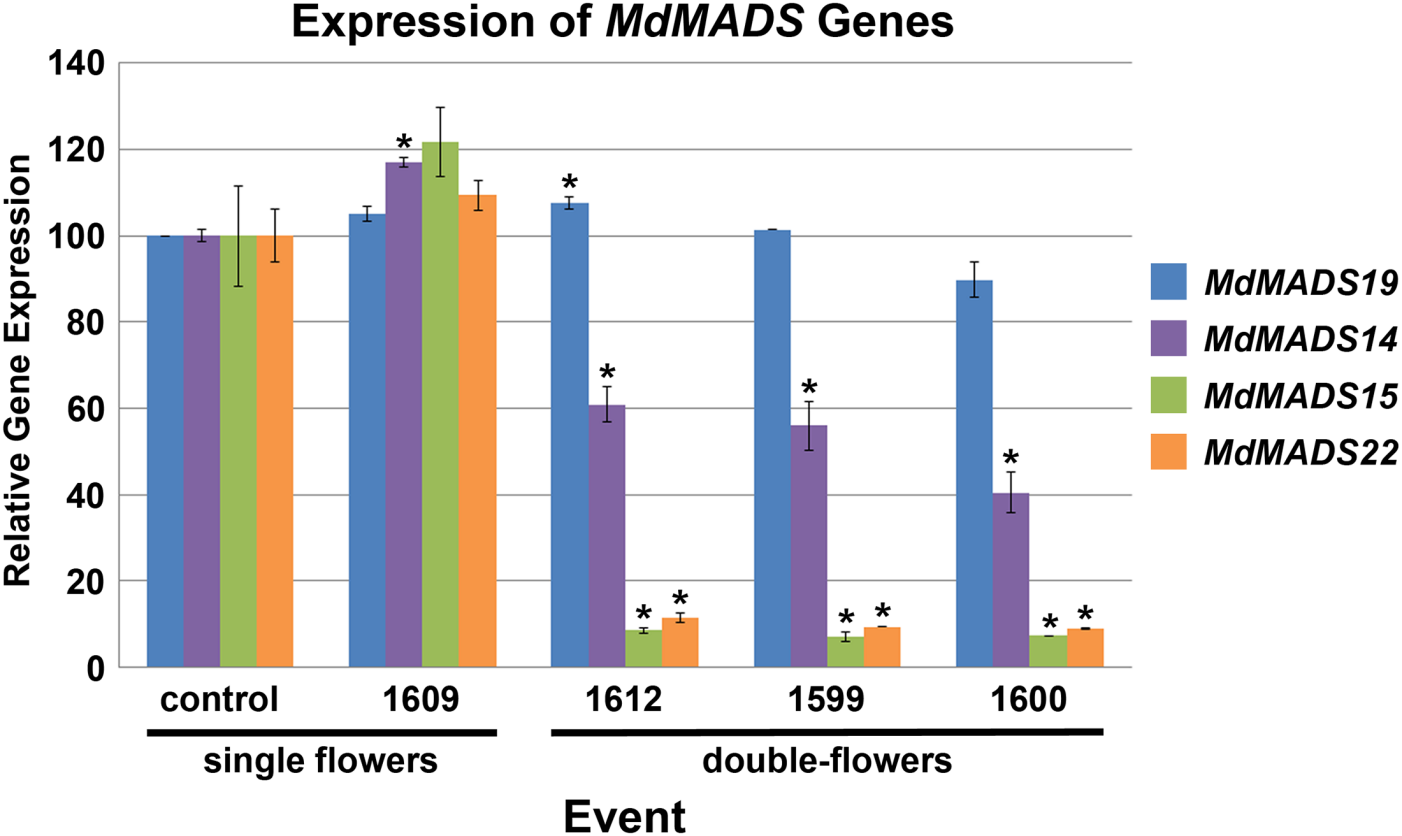
RNAi-*AG* double-flowers had decreased expression of *MdMADS22* and *MdMADS15*. The relative expression levels of *MdMADS19*, *MdMADS14*, *MdMADS22* and *MdMADS15* were quantified by qPCR for non-transgenic control trees, an event with single-flowers (1609), and three events with double-flowers (1599, 1600, and 1612). *MdMADS22* and *MdMADS15* were significantly reduced in all double-flower events, as was *MdMADS14*. Bars show standard error of the mean, asterisks indicate significant differences (P < .01).

We found that control trees and event 1609, which had single flowers, showed similar expression levels of all four *MdMADS* genes, except for a slight but significant increase in *MdMADS14* (P = .01, Fig 4). By contrast, events with double-flowers had significantly decreased expression of *MdMADS22* (9.9% of control expression, P < .01), and of *MdMADS15* (7.6% of control expression, P < .01*)*. *MdMADS14* expression was also significantly reduced in double-flowers (52.4% of control expression, P < .01). The amount of *MdMADS19* expression in all events was not significantly different from that of the control trees, with the exception of event 1612 showing a slight (7.5%) but significant increase in expression (P < .01).

### Fruit morphology

**Fig 5:**
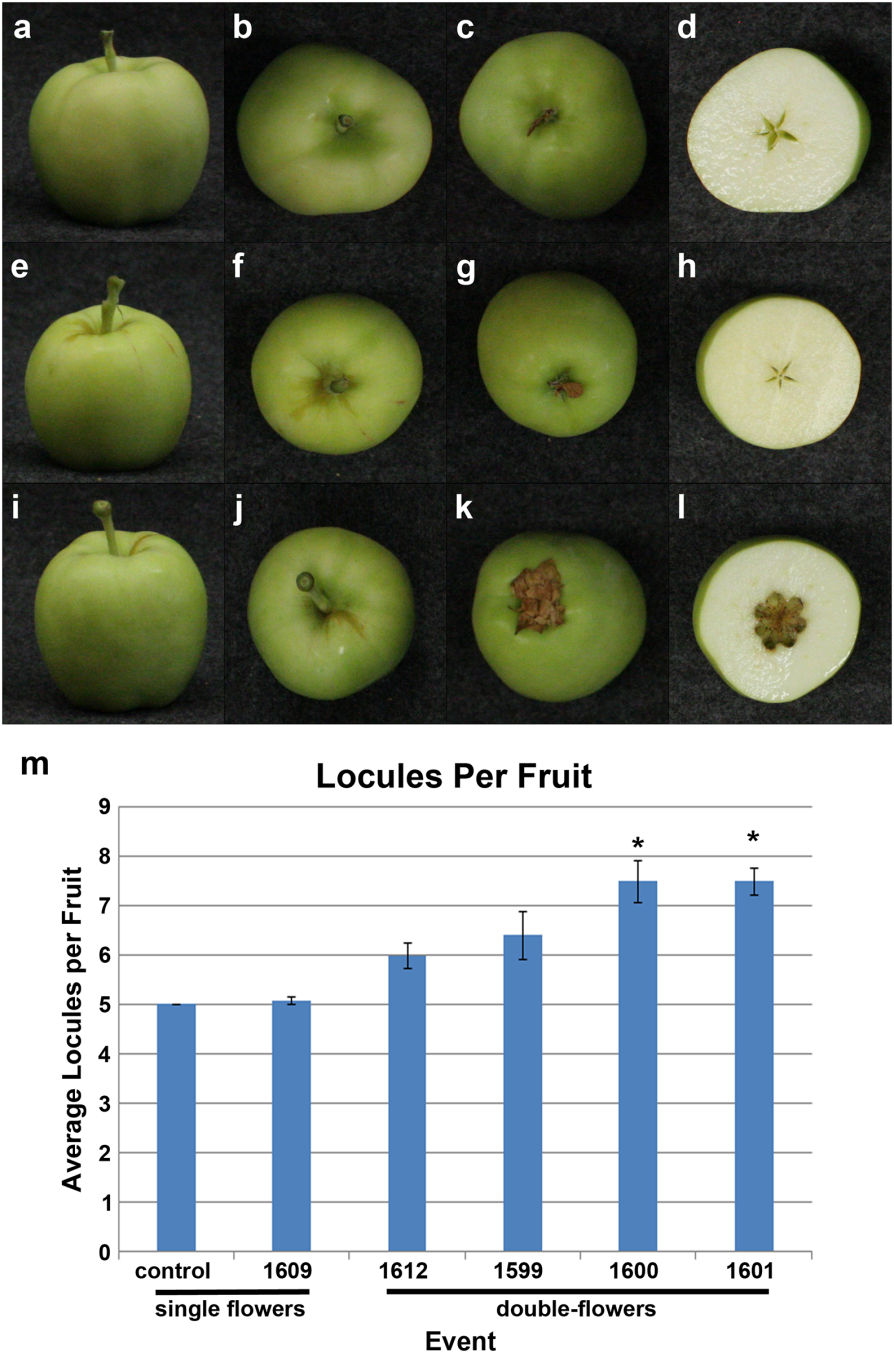
RNAi-*AG* trees with double-flowers set fruit with retained petaloid anthers and increased locules. (a-d) Fruits from control trees shed most of their petals and contained five narrow central compartments. (e-h) Fruits from single-flowered events were similar in overall appearance to control fruits, retained the occasional petal, and contained five internal compartments. (i-l) Fruits formed from double-flowers retained a large cluster of petaloid anthers on their base, had a large hollow in the center of the fruit, and an increased number of locules. (m) Quantification of the average number of locules per fruit showed that events with double-flowers (1600 and 1601) had significantly more locules than control fruits, while fruits from a single flowered event (1609) were similar to controls. All fruits shown were formed in the absence of cross pollination. Bars show standard error of the mean, asterisks indicate significant differences (P < .01).

Galaxy apple trees have the characteristic of setting fruit in the absence of pollination, providing an opportunity to analyze fruit morphology and seed content with and without cross-pollination. Analysis of parthenocarpic fruits showed that fruits from control and single-flowered trees shed most of their petals, and had an average of five internal compartments (Fig 5). By comparison, fruits developed from double-flowers retained a cluster of petaloid anthers which were highly-visible on the base of the fruit. Apart from this trait, the RNAi-*AG* fruits from double-flowers were, in general, externally similar to control fruits. Analysis of fruit interiors showed that fruits from double-flowers had a significantly increased number of enlarged but empty internal compartments (locules), which formed a hollow center (Fig 5, P < .05). Analysis of fruit size, as determined by weight, revealed that RNAi-*AG* fruits were full-sized or even slightly larger than fruits of control trees (S8 Fig, P < .05). Of the 44 fruits obtained from double-flowers, two fruits were found to have a fruit-in-a-fruit phenotype, with a small seed-containing structure inside of the main fruit cavity (S9 Fig). These two fruits also had enlarged stems. No such fruits were observed for control or single flowered RNAi-*AG* trees.

### Analysis of female fertility

**Fig 6:**
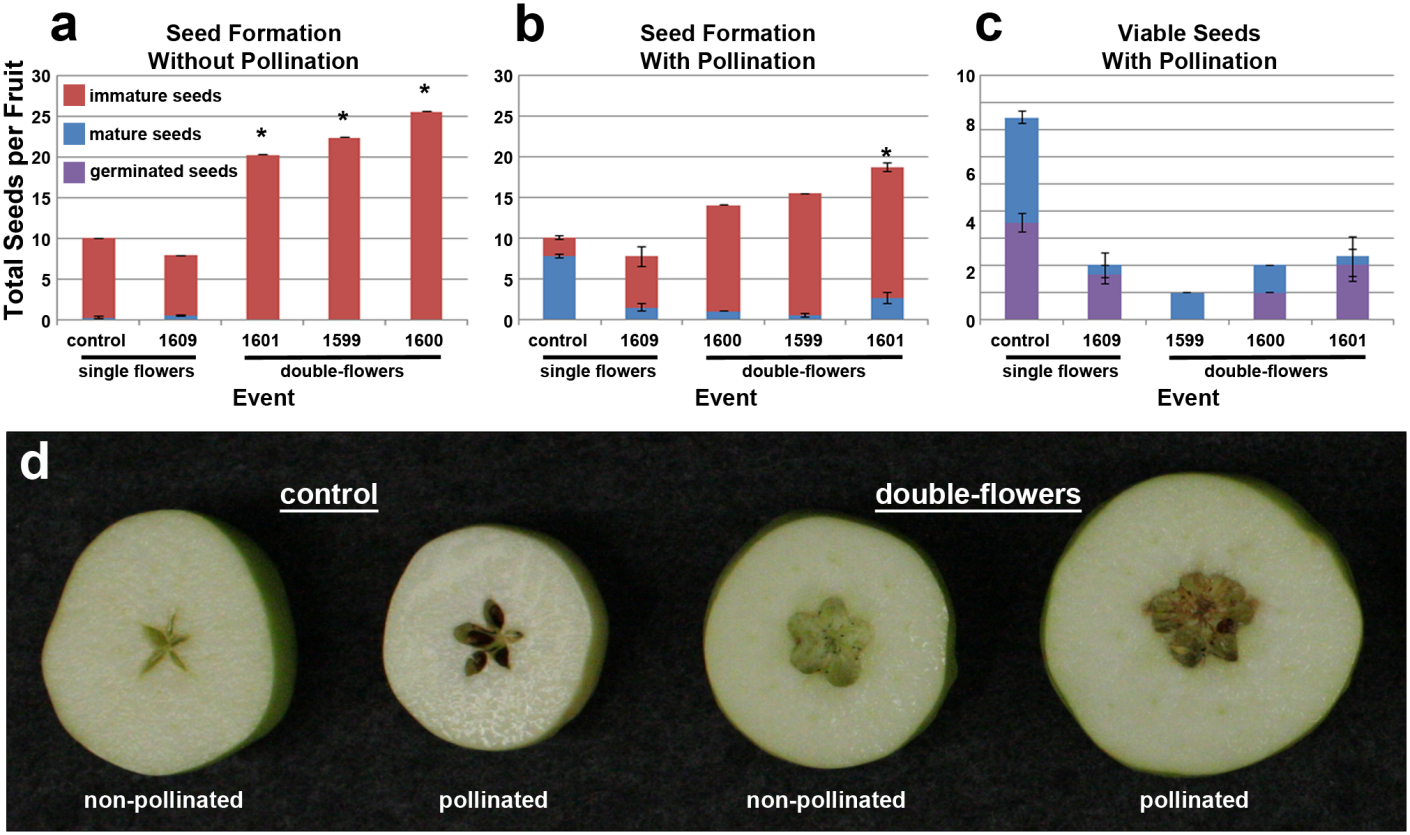
Fruit of double-flowers had few viable seeds after cross pollination. (a) Seed formation in the absence of cross pollination. (b) Seed formation with cross-pollination. (c) Germination mature seeds from cross-pollinated flowers. Bars show standard error over individual fruits. Asterisks show significant differences in total seeds as compared to control fruits (P < .01). (d) Examples of control and RNAi-*AG* fruits (from double-flowers) formed with and without cross-pollination.

In order to determine the effects of reduction of *MdMADS22* and *MdMADS15* on female fertility, we compared seed formation and seed viability in the presence and absence of cross-pollination. In the absence of cross-pollination, all fruits formed mostly immature seeds (Fig 6). On average, control fruits and fruits of RNAi-*AG* trees with single flowers had 10 total seeds, while RNAi-*AG* fruits from double-flowers had 20.3, 22.4, and 25.5 total seeds for events 1601, 1599, and 1600, respectively, with an overall average of 22.7 total seeds, a significant difference (P < .001). This increase in total seeds was due, in part, to the increase in the number of locules per fruit, and to an increase in the number of seeds per locule (Fig 5, S10 Fig). While control fruits typically had 2 seeds per locule, fruits from double-flowers had as many as 3.5 seeds per locule. After cross-pollination, control flowers formed fruits containing an average of 8 large, brown, mature seeds. By contrast, fruits of double-flowered RNAi-*AG* trees had an average of 0 to 2 mature seeds per fruit (S10 Fig). Viability testing of seeds formed after cross-pollination showed that control fruits had an average of 3.6 viable seeds per fruit, while fruits of double-flowered events averaged just 1 viable mature seed. Overall, despite the increase in total seeds, the fruit from double-flowers showed a 72% decrease in the number of viable seeds per fruit.

## Discussion

We found that RNAi-*AG* apple flowers had extra petals formed from the full to partial conversion of anthers to petals (Fig 1). This phenotype was consistent with reduction of AG function; petaloid anthers were previously reported as a result of RNAi of *AG* in *Arabidopsis thaliana* (*A*. *thaliana*) as well as tomato (*Solanum lycopersicum*), and these phenotypic changes were milder than the strong phenotype of *ag-1* allele in *A*. *thaliana* [14, 15]. Examination of gene expression from young apple flowers indicated that events with double-flowers had significantly decreased expression of both *MdMADS15* and *MdMADS22* (P < .01), demonstrating that RNAi can be used to target both of these *AG*-like genes simultaneously, as has been done with viral induced gene silencing in model species [16]. One advantage of RNAi is that it can be used to target multiple genes. However, it is also possible to suppress additional genes with sequence similarity to the primary target, as we observed for *MdMADS14* (Fig 4). A more distant MADS-box gene, *MdMADS19*, showed no reduction in expression in double-flowers. These results were consistent with the degree of DNA sequence similarity in the target region of the four genes, which were 99.8% (*MdMADS22*), 96.2% (*MdMADS15*), 68.5% (*MdMADS14*), and 63.3% (*MdMADS19*) identical to the sequence used for the RNAi construct. In addition, the longest regions of continuous identity in the more distant target genes *MdMADS14* and *MdMADS19* were only 16 and 18 base pairs (bp), respectively. RNAi is mediated by 21 bp RNAs [17] but is effective with mismatches at certain positions (reviewed in [18]). By comparison, the longest regions of continuous match for *MdMADS22* and *MdMADS15* were 414 bp and 153 bp, respectively, with hundreds of possible exact 21 bp matches across both of the sequences.

Our results add experimental support for a conserved role of *AG*-like genes in floral development of non-model species. Alterations in *AG* gene expression are associated with the formation of extra petals in a variety of plant species, including camellias (*Camelia*), ranuculids (*Ranunculus*), and other Rosaceous genera such as plums (*Prunus*) and roses (*Rosa*) [19–22]. In addition, while the floral phenotype exhibited by RNAi-*AG* apple trees was striking, it was not as strong as the phenotype of *ag* mutants of *A*. *thaliana*, where *ag-1* plants exhibit both a loss of anthers and carpels and a loss of floral meristem determinacy [8, 23, 24]. This finding may be a result of incomplete loss-of-function in our RNAi-*AG* apple trees. We found that double apple flowers had greatly-reduced, but not absent, levels of *MdMADS15* and *MdMADS22* (Fig 4), and the severity of RNAi-*AG* floral phenotypes are known to correlate with target gene mRNA levels [14]. Also, *MdMADS15* and *MdMADS22* are part of a large gene family, and there may be some functional overlap between genes. Other *MdMADS* family members may be fulfilling some *AG*-like function, possibly including the closely related *MdMADS14* gene—whose expression was only reduced by about half and which is similar to the *A*. *thaliana SHP* genes (S Fig 1). *A*. *thaliana SHP* genes have some functional overlap with *AG* in carpel formation [25]. It is possible that *MdMADS14* performs similarly in apple, leading to a mild phenotype.

We found that the double-flowers of RNAi-*AG* apple trees had reductions in both male and female fertility, but with male fertility more affected, in regards to the severity of floral organ morphology, than female fertility (Figs 1–3). The physical conversion of anthers to petals greatly decreased the production of pollen grains of double-flowers as compared to control flowers (Fig 2). While the innermost petaloid anthers retained a more anther-like morphology, both the amount and the viability of pollen grains were reduced (Fig 2). Also, these pollen grains were physically enclosed within the organ and were not released. It is possible that the decrease in pollen grain formation was due to suppression of *MdAG*-like genes *MdMADS15* and *MdMADS22*, as *AG* induces pollen formation in *A*. *thaliana* [26]. The combination of grain retention, decreased pollen production, and reduced pollen viability caused a large loss of male reproductive capacity for the double-flowers.

Alterations in the female reproductive capacity of the double-flowered RNAi-*AG* trees were complex. These findings were a result of the contrasting effects of RNAi-*AG* on the production of stigmas versus seed formation. Quantification of the number of stigmas revealed a significant decrease in the average number of stigmas per flower for events 1600 and 1612 (Fig 3, P < .01), and that many of the styles had projections of petal-like tissue, indicating they had undergone a partial organ identity conversion to non-reproductive structures. However, double-flowers produced far more total seeds than control flowers (Fig 6). This increase was due to both the presence of additional internal compartments, and to an increase in the number of seeds per compartment (Fig 5, S10 Fig). The formation of extra compartments and seeds may have been due to a reduction in floral meristem determinacy of the double-flowers, as may the observation of an occasional apple-in-apple phenotype (S6 Fig). These fruit-in-a-fruit apples also had noticeably larger stems than control apples. Despite this increase in total seed number, double-flowers had fewer mature seeds following cross-pollination than did control flowers (Fig 6). This reduction was probably due to a combination of factors, including modification of stigma and style structures, which may be less supportive of pollen tube growth than control structures. The reduction in mature seed formation in fruits formed from double-flowers may have also been due, in part, to the reduction in *MdMADS14* expression. *MdMADS14* is similar to the *A*. *thaliana SHP* genes (S1 Fig), which have functions in ovule development [25]. The observation that the double-flowers still retained some pollen production and seed formation indicated that complete sterility was not achieved for these trees. For genetic containment purposes, a complete loss of fertility is likely needed [27]. We predict that the production and analysis of a larger group of RNAi events, or the use of site-directed mutagenesis methods such as CRISPR/Cas9, which causes physical alterations in target DNA sequences (reviewed in [28]), would lead to a much stronger phenotype and complete male and female sterility. Full loss of AG function may require the complete knock-out of both *MdMADS15* and *MdMADS22* (S1 Fig). It is likely that apple trees completely lacking in *AG* function would be fruitless, as carpel tissues would no longer be present.

In addition to genetic containment and enhanced floral display, apples produced with partial loss of *AG* function may have other benefits. If the cultivar produced fruits in the absence of pollination, the resulting seedless fruits could be decorative, or serve as a source of food for wildlife. Also, apple trees with female sterility would lack mature seeds, which may be a benefit to the juice and cider industries, as apple seeds contain unwanted compounds, including cyanides [29, 30]. Consumers may also find seedless apples appealing, although the core is likely to still be present. Commercial varieties of apple trees are propagated asexually by vegetative cuttings [31, 32], meaning that a loss of seed production would not be an impediment to tree multiplication. The main near-term value of these trees would be in reduction of unwanted pollen transfer from GE to traditional trees. Apple trees are bee pollinated, and GE trees grown in proximity to non-GE trees can have their pollen transferred to these neighboring trees, sometimes at a considerable distance [33].

While RNAi of *MdAG*-like genes led to dramatic alterations in floral form, unlike other *MADS*-suppressed apples [34], RNAi-*AG* fruits were nearly normal in morphology and were equally as large, or larger, than fruits from control trees (S5 Fig). This characteristic was likely highly influenced by use of the Galaxy cultivar, which sets fruits in the absence of pollination. Other cultivars with suppressed *AG* may be fruitless, and thus could be grown for their decorative flowers, and may be useful in locations where fruits would be considered a nuisance. RNAi-*AG* fruits had some distinctive features not found on control fruits, such as retaining a large cluster of dried petals on the base of the fruit, and hollow interiors. Such features would make it possible to readily identify intact fruits produced by double-flowered trees, which could be useful as a means to track them. The lack of mature seeds should serve to help extend the shelf life of the fruits (reviewed in [35]). However, it is also possible that the hollow interior formed in fruit from double-flowers may negatively impact fruit quality or shelf life; it would be important to study these traits as part of further product development.

The recent deregulation of the Arctic^™^ apple and Innate^™^ potatoes [36, 37] indicates that there may be growing uptake of GE varieties of asexually propagated plants. New varieties of apples with resistance to apple scab or with red-fleshed fruits may be valued by producers and consumers, and find their way on to the market [38, 39]. In some areas, the addition of containment technology may facilitate uptake and coexistence.

The attractive floral phenotype of RNAi-*AG* apples, and *Rosaceous* ornamentals with similar morphology, together with reduced production of allergenic pollen, may make them highly desirable for ornamental plantings. Many-petaled varieties of trees are popular with consumers, such as flowering cherry (*Prunus serrulata* ‘Kanzan’) and ornamental crabapple (*Malus* ‘Branzam’). Likewise, male-sterile or female-only (dioecious) varieties of trees are popular with allergy-prone consumers for some varieties of ornamental species and geographies [40]. The ability to modify popular varieties to make them more attractive, less allergenic, and with less risk of becoming invasive, might help RNAi or site-directed mutagenesis technology to gain acceptance in the ornamentals industry.

## Materials and Methods

### Vector construction and plant growth

Total RNA was isolated from spring floral buds of local Fuji apple trees using a modified RNeasy Plant Mini kit (Qiagen) and used for the synthesis of cDNA using Superscript I reverse transcriptase (Invitrogen). A 420 bp fragment of apple *AG*-like cDNA was cloned in sense and antisense orientations in the pHannibal vector creating an inverted repeat flanking an intron. The resulting 35S:MdAGIR:OCSterm was inserted into the binary vector pART27 (S2 Fig) and used for transformation of *Malus domestica* cultivar Galaxy using published methods [41]. Transformed events were verified by PCR and grafted onto a G.11 dwarfing rootstock in 2005. Grafted trees were grown in a greenhouse for eight years at the New York State Agricultural Experiment Station (Cornell University) in Geneva, New York. During the following dormant season (2014), trees representing five events, along with non-transgenic controls, were shipped as bare-root trees in April 2014 to Oregon State University in Corvallis, Oregon. Trees were potted, randomized, and grown in a greenhouse with supplemental lighting for two additional years, where they were sampled for gene expression and phenotypic analyses from April 2014 until December of 2014.

### Sequencing of portions of *MdMAD*S genes

Transcripts for each of the four selected *MdMADS* genes were sequenced from Galaxy control trees using primers specific to *MdMADS22* (5’-ATTTGTGGAGCTGTGAAAAAA-3’) and (5’-TTTCACATGTAAGTTGGAGGAAC-3’), *MdMADS15* (5’–GGCCTATGAAAGCAAATCCTTGT-3’) and (5’–TCTTCATTCCCCAAGCAATCTG-3’), *MdMADS14* (5’–GACAAGTCACCTTCTGCAAAC-3’) and (5’–CGTCAATTGTCTCATAGATTTATGAAG-3’), and *MdMADS19* (5’–ATAAGCTTTGGCTTCTTTGGG-3’) (5’–GGGTAGTACTGGGTACTAAACC-3’). Percent identities of sequenced gene regions and the RNAi hairpin sequence were calculated using CLUSTALW2 [42].

### Analysis of floral morphology

Trees were monitored for flower formation each spring. Flowers were imaged using a Canon Rebel XSI digital camera. The average number of stigmas per flower was determined by counting the number of stigmas present in 30 total flowers from three trees (ten flowers per tree, from two clusters of 5 flowers chosen for similar amounts of floral opening) from each event, and from three control trees. A t-test was used to determine if the number of stigmas per flower were significantly different. Representative flowers were dissected and the floral organs imaged using a Keyence VHX-1000 digital microscope. Anthers and petaloid anthers were cross sectioned for imaging of pollen grains. Organs of flowers used for floral montages were removed and placed in order of phylotaxy, beginning with the sepals and ending with the gynoecium.

### Pollen staining and quantification

Mature flowers were collected and fixed in a 6:3:1 ethanol:chloroform:acetic acid solution. Control pollen grains were actively shedding at the time of floral collection and were obtained by pipetting fixative and grains from the bottom of the collection tube. Pollen from double-flowers was obtained by dissecting fixed petaloid anthers and dabbing the cut sections onto a microscope slide. Pollen grains were stained using a simplified Alexander staining protocol [43]. Slides were sealed with nail polish and immediately viewed using a Keyence VHX-1000 digital microscope. Pollen from control trees, one single-flowered RNAi-*AG* event, and four double-flowered RNAi-*AG* events was stained. Pollen grain viability was quantified for at least 130 pollen grains per event, collected from at least 3 non-overlapping fields of view. A Chi-squared test was used to determine if the numbers of viable and non-viable pollen grains per event were significantly different from those of control trees.

### Phylogenetic analysis of apple *MdMADS* genes

The *Arabidopsis thaliana AG*, *STK* and *SHP* proteins were used for BLASTP queries of the Phytozyme9.1 database to identify AG family members in the genomes of *Malus domestica*, *Prunus persica*, *Fragaria vesca*, *Populus trichocarpa*, *Vitis vinifera* (Phytozyme10.1 database) and *Pyrus x bretschneideri* (NCBI Pbr v1.0 reference, Annotation release 100). Family members from other species were obtained by searching the NCBI protein database. For *Malus*, proteins predicted from full-length cDNAs were also obtained and in two cases used in place of proteins predicted from the genome sequence (see S2 Table for a detailed description and for all gene IDs). Sequence alignment was produced using MUSCLE [44]. Phylogenetic analysis was performed using the neighbor-joining tree method in the Mega 6.0 computer program [45]. One-thousand bootstrapped data sets were used to infer consensus trees and associated estimates of statistical confidence.

### Gene expression analysis

Total RNA was isolated using a rapid CTAB-based RNA extraction method [46] from whole young flower buds collected April 2014 and immediately frozen in liquid nitrogen. RNA was treated with DNase (DNase I, Amplification Grade, Invitrogen) according to the manufacturer’s protocol. SuperScript III Reverse Transcriptase (Invitrogen) was used for cDNA synthesis following the manufacturer’s recommendation. Quantitative PCR (qPCR) was performed using an Applied Biosystems StepOnePlus real-time PCR system (Applied Biosystems). The housekeeping gene *ACTIN* (*ACT*) was used as a reference and three technical replicates were used for each reaction. The following gene specific primers were used: *ACT* (5’-GGACAGCGAGGACATTCAGC-3’) and (5’-CTGACCCATTCCAACCATAACA-3’), *MdMADS19* (5’-GGAGATTGAGCTGGAAAACG-3’) and (5’-CTCCTCCACCCTCGATCATA-3’), *MdMADS14* (5’-CGACAAGTCACCTTCTGCAA-3’) and (5’- ATCCACCGTCCGTAGAATCA-3’), *MdMADS15* (5’-GGGTAGGGGAAGATCGAGATC-3’) and (5’-CCAGTATTTGAAGAATCTGCACATGT-3’), *MdMADS22* (5’-GGCTGGAGGAGGAAGCTATGAG-3’) and (5’-GTGATGATTGGGTTGTAACA-3’). Reactions contained 7.5 μl SYBR Green mix (Platinum^®^ SYBR^®^ Green qPCR SuperMix-UDG, Invitrogen), 10 ng cDNA, 0.3 μl of forward and reverse primers (10 μM each), 0.3 μl ROX reference dye, and water for a total reaction volume of 15 μl. The PCR program consisted of 3 min at 95°C followed by 40 cycles of 20s at 95°C, 20s at 58°C and 20s at 72°C, followed by melt curve analysis: 15s at 95°C, 60s at 58°C, then elevated 0.2°C per second to 95°C. StepOne Software version 2.2 (Applied Biosystems) was used for data analysis. Data shown are from one representative experiment. Each bar is based on the average and variation among three biological replicates (individual flower buds collected from different trees) run in triplicate reactions. A Student’s t-test was used to determine if expression levels were significantly different between control and RNAi-*AG* samples.

### Cross pollination and analysis of fruit morphology

Non-transgenic apple pollen (variety Idared) was used to hand-pollinate selected flowers, pollinated clusters were labeled and monitored for fruit formation. All apples retained on trees were collected, sorted by tree and by category (apples from non-pollinated flowers, apples from cross-pollinated flowers), weighed, and photographed using a Canon Rebel XSI digital camera. Fruits were hand-sectioned with a sharp knife to examine internal fruit morphology and to count the seeds present in each fruit. Seeds were classified as developed (large, plump) or undeveloped (small, shriveled) based on visual inspection. Cross-sectioned fruits were used to count the numbers of locules present in each apple. Sectioned fruit pieces were dipped in a 3% citric acid solution to delay browning prior to photography. Once fruits were photographed, seeds were removed and retained for germination testing. T-tests were used to determine if the average fruit weights, seed numbers, seeds per locule, and locules per fruit were different between controls and transgenic events.

### Seed germination

Seeds were collected, air dried, and vernalized (4°C for 6 weeks) on damp filter paper in sealed Petri dishes at 4°C. Both developed (large, plump) and undeveloped (small, shriveled) seeds were tested. Seeds were checked periodically for mold formation, which was removed by gently blotting with Kimwipes, and placed on fresh damp filter paper as needed. Following vernalization, seeds were placed on fresh damp filter paper in new sealed Petri dishes under constant illumination. Seeds were scored as viable if they developed a root of at least 1 cm in length within 14 days.

## Supporting Information

S1 Fig Phylogeny depicting relationships of *Malus* members of the *AG* subfamily. Alignment of protein sequences and the neighbor-joining method were used to produce the tree, and bootstrap values of 50% or higher are indicated at the nodes. Clades are named based on the *Arabidopsis thaliana* members. All family members identified in the *Malus x domestica* (prefix Md, shown in red) *Prunus persica* (Prupe), *Pyrus x bretschneideri* (Pbr), *Fragaria vesca* (Fv), *Populus trichocarpa* (Potri), and *Vitis vinifera* (Vv) genomes were included. Additional proteins were from *Antirrhinum majus* (PLE, FAR) *Solanum lycoperscium* (ALQ, TAG1, TAGL11), *Petunia x hybrida* (PMADS3, FBP7, FBP11).
(TIF)

S2 Fig Diagram of construct used for apple transformation. An inverted repeat of a 420 bp fragment of the apple *MdMADS22* gene (*MdAG*) was cloned into the pART27 vector under control of the constitutive 35S promoter and terminated by the octapine synthase 3’ untranslated region (tOCS). This construct contained a neomycin phosphotransferase II (nptII) gene controlled by the constitutive nopaline synthase promoter (pNOS) and terminated by the nopaline synthase 3’ untranslated region (tNOS). Arrows indicate the direction of transcriptional activation. The left border and right border are indicated by LB and RB, respectively.
(TIF)

S3 Fig Outer petals of double-flowers were normal in appearance. (a) Control trees had five total petals (b) while double-flowers had five outer petals and numerous inner petals. These five outermost petals were highly similar to petals of control trees and opened before the inner petals.
(TIF)

S4 Fig Alignment of RNAi targeting sequence with *MdMADS*22. Alignment of the sequence used to target apple *AG*-like genes with *MdMADS22*. Perfect matches are indicated by asterisks below the matched bases, dashes indicate gaps, and numbers indicate base pairs.
(TIF)

S5 Fig Alignment of RNAi targeting sequence with *MdMADS*15. Alignment of the sequence used to target apple *AG*-like genes with *MdMADS15*. Perfect matches are indicated by asterisks below the matched bases, dashes indicate gaps, and numbers indicate base pairs.
(TIF)

S6 Fig Alignment of RNAi targeting sequence with *MdMADS14*. Alignment of the sequence used to target apple *AG*-like genes with *MdMADS14*. Perfect matches are indicated by asterisks below the matched bases, dashes indicate gaps, and numbers indicate base pairs.
(TIF)

S7 Fig Alignment of RNAi targeting sequence with *MdMADS19*. Alignment of the sequence used to target apple *AG*-like genes with *MdMADS19*. Perfect matches are indicated by asterisks below the matched bases, dashes indicate gaps, and numbers indicate base pairs.
(TIF)

S8 Fig RNAi-*AG* trees set full-sized fruit. Quantification of fruit size (by weight) showed that fruits produced by RNAi events were as large as fruits produced by control trees. Bars show standard error of the mean.
(TIF)

S9 Fig Some RNAi-*AG* trees set fruit with an apple-in-apple phenotype. (a) Apples from control trees contained a hollow internal cavity with small undeveloped seeds (c, arrow). (b) Two of the RNAi-*AG* events set fruit with a large fleshy structure in the center that contained small undeveloped seeds (d, arrow).
(TIF)

S10 Fig RNAi-*AG* apples from double-flowers had an increased number of seeds per locule. Fruits developed from RNAi-*AG* events with double-flowers had an increased number of seeds per locule. Bars show standard error, asterisks indicate significant differences (P < .01).
(TIF)

S1 Table Floral phenotypes by event. Eight independent events represented by 1–16 individual ramets (trees) were tested. Flowers were classified as either single (phenotypically similar to non-transgenic control flowers) or double (possessing extra petals).
(TIF)

S2 Table Genes and gene models used in phylogenetic analysis. The names, gene ID(s), GenBank cDNA accessions, and species for all genes used in the phylogenetic analysis are shown. Three of the *MdMADS* genes had gene IDs which differed from the GenBank cDNA accessions. Specifically, the *MdMADS10* gene ID had two partial gene IDs associated with it. The MDP0000268317 prediction missed calling 3' exons and model MDP0000306884 lacks a MADS-box, likely due to the sequence being located at the end of a scaffold. The cDNA encoded protein was used for phylogenetic analysis. The gene ID for *MdMADS25* predicted different (atypical) splicing compared to the cDNA sequence; the cDNA encoded protein was used for phylogenetic analysis. None of the current gene IDs for *MdMADS19* matched the GenBank cDNA accession. A combination of alternate models MDP0000061338 and MDP0000331635 resulted in a best fit to the cDNA accession for this gene, na, not applicable.
(TIF)

## References

[1] APHIS. Questions and answers: Arctic apple deregulation. In: Agriculture USDo, editor. 2015.

[2] Vining KJ, Contreras RN, Ranik M, Strauss SH. Genetic Methods for Mitigating Invasiveness of Woody Ornamental Plants: Research Needs and Opportunities. Hortscience. 2012;47(9):1210–6. ISI:000309032900002.

[3] Ranney TG. Population Control: Developing Non-Invasive Nursery Crops©. Combined Proceedings International Plant Propagators’ Society. 2004;54:604–7.

[4] Elorriaga E, Meilan R, Ma C, Skinner JS, Etherington E, Brunner A, et al A tapetal ablation transgene induces stable male sterility and slows field growth in Populus. Tree Genet Genomes. 2014;10(6):1583–93. 10.1007/s11295-014-0781-6. ISI:000343924600008.

[5] Zhang C, Norris-Caneda KH, Rottmann WH, Gulledge JE, Chang S, Kwan BY, et al Control of pollen-mediated gene flow in transgenic trees. Plant Physiol. 2012;159(4):1319–34. Epub 2012/06/23. 10.1104/pp.112.197228 22723085 PMC3425181

[6] Klocko AL, Brunner AM, Huang J, Meilan R, Lu H, Ma C, et al Containment of transgenic trees by suppression of LEAFY. Nat Biotechnol. 2016;in press. 10.1038/nbt.3636 27606454

[7] Culley TM, Hardiman NA. The beginning of a new invasive plant: A history of the ornamental Callery pear in the United States. Bioscience. 2007;57(11):956–64. 10.1641/B571108. ISI:000251871800008.

[8] Bowman JL, Smyth DR, Meyerowitz EM. Genes directing flower development in Arabidopsis. Plant Cell. 1989;1(1):37–52. Epub 1989/01/01. 10.1105/tpc.1.1.37 2535466 PMC159735

[9] Bowman JL, Smyth DR, Meyerowitz EM. Genetic interactions among floral homeotic genes of Arabidopsis. Development. 1991;112(1):1–20. Epub 1991/05/01. . 1685111 10.1242/dev.112.1.1

[10] Dreni L, Kater MM. MADS reloaded: evolution of the AGAMOUS subfamily genes. New Phytol. 2014;201(3):717–32. Epub 2013/10/30. 10.1111/nph.12555 . 24164649

[11] van der Linden CG, Vosman B, Smulders MJ. Cloning and characterization of four apple MADS box genes isolated from vegetative tissue. Journal of Experimental Botany. 2002;53(371):1025–36. Epub 2002/04/25. . 11971914 10.1093/jexbot/53.371.1025

[12] Velasco R, Zharkikh A, Affourtit J, Dhingra A, Cestaro A, Kalyanaraman A, et al The genome of the domesticated apple (Malus x domestica Borkh.). Nature Genetics. 2010;42(10):833–9. Epub 2010/08/31. 10.1038/ng.654 . 20802477

[13] Tian Y, Dong Q, Ji Z, Chi F, Cong P, Zhou Z. Genome-wide identification and analysis of the MADS-box gene family in apple. Gene. 2014:277–90. Epub 2014/12/03. 10.1016/j.gene.2014.11.018 . 25447908

[14] Chuang CF, Meyerowitz EM. Specific and heritable genetic interference by double-stranded RNA in Arabidopsis thaliana. Proc Natl Acad Sci U S A. 2000;97(9):4985–90. Epub 2000/04/26. 10.1073/pnas.060034297 10781109 PMC18344

[15] Pan IL, McQuinn R, Giovannoni JJ, Irish VF. Functional diversification of AGAMOUS lineage genes in regulating tomato flower and fruit development. Journal of Experimental Botany. 2010;61(6):1795–806. Epub 2010/03/26. 10.1093/jxb/erq046 20335407 PMC2852668

[16] Fourquin C, Ferrandiz C. Functional analyses of AGAMOUS family members in Nicotiana benthamiana clarify the evolution of early and late roles of C-function genes in eudicots. Plant J. 2012;71(6):990–1001. Epub 2012/05/09. 10.1111/j.1365-313X.2012.05046.x . 22563981

[17] Elbashir SM, Lendeckel W, Tuschl T. RNA interference is mediated by 21-and 22-nucleotide RNAs. Gene Dev. 2001;15(2):188–200. 10.1101/Gad.862301. ISI:000166683800007. 11157775 PMC312613

[18] Ahmed F, Raghava GPS. Designing of Highly Effective Complementary and Mismatch siRNAs for Silencing a Gene. PLoS One. 2011;6(8):e23443 ARTN e23443 10.1371/journal.pone.0023443. ISI:000295454200086. 21853133 PMC3154470

[19] Dubois A, Raymond O, Maene M, Baudino S, Langlade NB, Boltz V, et al Tinkering with the C-function: a molecular frame for the selection of double flowers in cultivated roses. PLoS One. 2010;5(2):e9288 Epub 2010/02/23. 10.1371/journal.pone.0009288 20174587 PMC2823793

[20] Galimba KD, Tolkin TR, Sullivan AM, Melzer R, Theissen G, Di Stilio VS. Loss of deeply conserved C-class floral homeotic gene function and C- and E-class protein interaction in a double-flowered ranunculid mutant. Proc Natl Acad Sci U S A. 2012;109(34):E2267–75. Epub 2012/08/03. 10.1073/pnas.1203686109 22853954 PMC3427126

[21] Liu Z, Zhang D, Liu D, Li F, Lu H. Exon skipping of AGAMOUS homolog PrseAG in developing double flowers of Prunus lannesiana (Rosaceae). Plant Cell Rep. 2013;32(2):227–37. Epub 2012/10/26. 10.1007/s00299-012-1357-2 . 23096754

[22] Sun Y, Fan Z, Li X, Liu Z, Li J, Yin H. Distinct double flower varieties in Camellia japonica exhibit both expansion and contraction of C-class gene expression. BMC Plant Biol. 2014;14(1):288 Epub 2014/10/26. 10.1186/s12870-014-0288-1 25344122 PMC4219040

[23] Koornneef J, Bruin d, Goettsch P. A provisional map of chromosome 4 of Arabidopsis. Arabidopsis Information Service. 1980;17:11–8.

[24] Koornneef M, Vaneden J, Hanhart CJ, Stam P, Braaksma FJ, Feenstra WJ. Linkage Map of Arabidopsis-Thaliana. J Hered. 1983;74(4):265–72. ISI:A1983QZ18300007.

[25] Pinyopich A, Ditta GS, Savidge B, Liljegren SJ, Baumann E, Wisman E, et al Assessing the redundancy of MADS-box genes during carpel and ovule development. Nature. 2003;424(6944):85–8. Epub 2003/07/04. 10.1038/nature01741 . 12840762

[26] Ito T, Wellmer F, Yu H, Das P, Ito N, Alves-Ferreira M, et al The homeotic protein AGAMOUS controls microsporogenesis by regulation of SPOROCYTELESS. Nature. 2004;430(6997):356–60. Epub 2004/07/16. 10.1038/nature02733 . 15254538

[27] Knight TM, Havens K, Vitt P. Will the Use of Less Fecund Cultivars Reduce the Invasiveness of Perennial Plants? Bioscience. 2011;61(10):816–22. 10.1525/bio.2011.61.10.11. ISI:000299805400011.

[28] Sander JD, Joung JK. CRISPR-Cas systems for editing, regulating and targeting genomes. Nat Biotechnol. 2014;32(4):347–55. Epub 2014/03/04. 10.1038/nbt.2842 24584096 PMC4022601

[29] Barceloux DG. Cyanogenic Foods (Cassava, Fruit Kernels, and Cycad Seeds) Medical Toxicology of Natural Substances: Foods, Fungi, Medicinal Herbs, Toxic Plants, and Venomous Animals. Hoboken, JH: John Wiley & Sons; 2008.

[30] Bolarinwa IF, Orfila C, Morgan MRA. Determination of amygdalin in apple seeds, fresh apples and processed apple juices. Food Chem. 2015;170:437–42. 10.1016/j.foodchem.2014.08.083. ISI:000343780400059. 25306368

[31] Shulaev V, Korban SS, Sosinski B, Abbott AG, Aldwinckle HS, Folta KM, et al Multiple models for Rosaceae genomics. Plant Physiol. 2008;147(3):985–1003. Epub 2008/05/20. 10.1104/pp.107.115618 18487361 PMC2442536

[32] Nybom H, Rogstad SH, Schaal BA. Genetic variation detected by use of the M13 "DNA fingerprint" probe in Malus, Prunus, and Rubus (Rosaceae). Theor Appl Genet. 1990;79(2):153–6. Epub 1990/02/01. 10.1007/BF00225944 . 24226211

[33] Tyson RC, Ben Wilson J, Lane WD. A mechanistic model to predict transgenic seed contamination in bee-pollinated crops validated in an apple orchard. Ecol Model. 2011;222(13):2084–92. 10.1016/j.ecolmodel.2011.03.039. ISI:000292581400006.

[34] Ireland HS, Yao JL, Tomes S, Sutherland PW, Nieuwenhuizen N, Gunaseelan K, et al Apple SEPALLATA1/2-like genes control fruit flesh development and ripening. Plant J. 2013;73(6):1044–56. Epub 2012/12/15. 10.1111/tpj.12094 . 23236986

[35] Pandolfini T. Seedless fruit production by hormonal regulation of fruit set. Nutrients. 2009;1(2):168–77. Epub 2009/02/01. 10.3390/nu1020168 22253976 PMC3257607

[36] Waltz E. Nonbrowning GM apple cleared for market. Nat Biotechnol. 2015;33(4):326–7. 10.1038/nbt0415-326c. ISI:000352348500009. 25850045

[37] Waltz E. USDA approves next-generation GM potato. Nat Biotechnol. 2015;33(1):12–3. 10.1038/nbt0115-12. ISI:000347714200013. 25574623

[38] Krens FA, Schaart JG, van der Burgh AM, Tinnenbroek-Capel IE, Groenwold R, Kodde LP, et al Cisgenic apple trees; development, characterization, and performance. Frontiers in Plant Science. 2015;6:286 Epub 2015/05/13. 10.3389/fpls.2015.00286 25964793 PMC4410516

[39] Vanblaere T, Flachowsky H, Gessler C, Broggini GA. Molecular characterization of cisgenic lines of apple 'Gala' carrying the Rvi6 scab resistance gene. Plant Biotechnology Journal. 2014;12(1):2–9. Epub 2013/09/04. 10.1111/pbi.12110 . 23998808

[40] Ogren TL. Allergy-Free Gardening: The Revolutionary Guide to Healthy Landscaping: Ten Speed Press; 2004.

[41] Boresjza-Wysocka E, Norelli JL, Ko K, Aldwinckle HS. Transformation of authentic M.26 apple rootstock for enhanced resistance to fire blight. Acta Horticulture. 1999;489:259–66.

[42] Larkin MA, Blackshields G, Brown NP, Chenna R, McGettigan PA, McWilliam H, et al Clustal W and Clustal X version 2.0. Bioinformatics. 2007;23(21):2947–8. Epub 2007/09/12. 10.1093/bioinformatics/btm404 . 17846036

[43] Peterson R, Slovin JP, Chen C. A simplified method for differential staining of aborted and non-aborted pollen grains. International Journal of Plant Biology. 2010;1(2):e13.

[44] Edgar RC. MUSCLE: multiple sequence alignment with high accuracy and high throughput. Nucleic Acids Research. 2004;32(5):1792–7. Epub 2004/03/23. 10.1093/nar/gkh340 15034147 PMC390337

[45] Tamura K, Stecher G, Peterson D, Filipski A, Kumar S. MEGA6: Molecular Evolutionary Genetics Analysis version 6.0. Mol Biol Evol. 2013;30(12):2725–9. Epub 2013/10/18. 10.1093/molbev/mst197 24132122 PMC3840312

[46] Gambino G, Perrone I, Gribaudo I. A Rapid and effective method for RNA extraction from different tissues of grapevine and other woody plants. Phytochem Anal. 2008;19(6):520–5. Epub 2008/07/12. 10.1002/pca.1078 . 18618437

