# Supplementary figures and images for: Transgenic Suppression of AGAMOUS Genes in Apple Reduces Fertility and Increases Floral Attractiveness
Source: PLoS One. 2016 Aug 8;11(8):e0159421. doi: 10.1371/journal.pone.0159421 (PMC4976969; doi:10.1371/journal.pone.0159421)

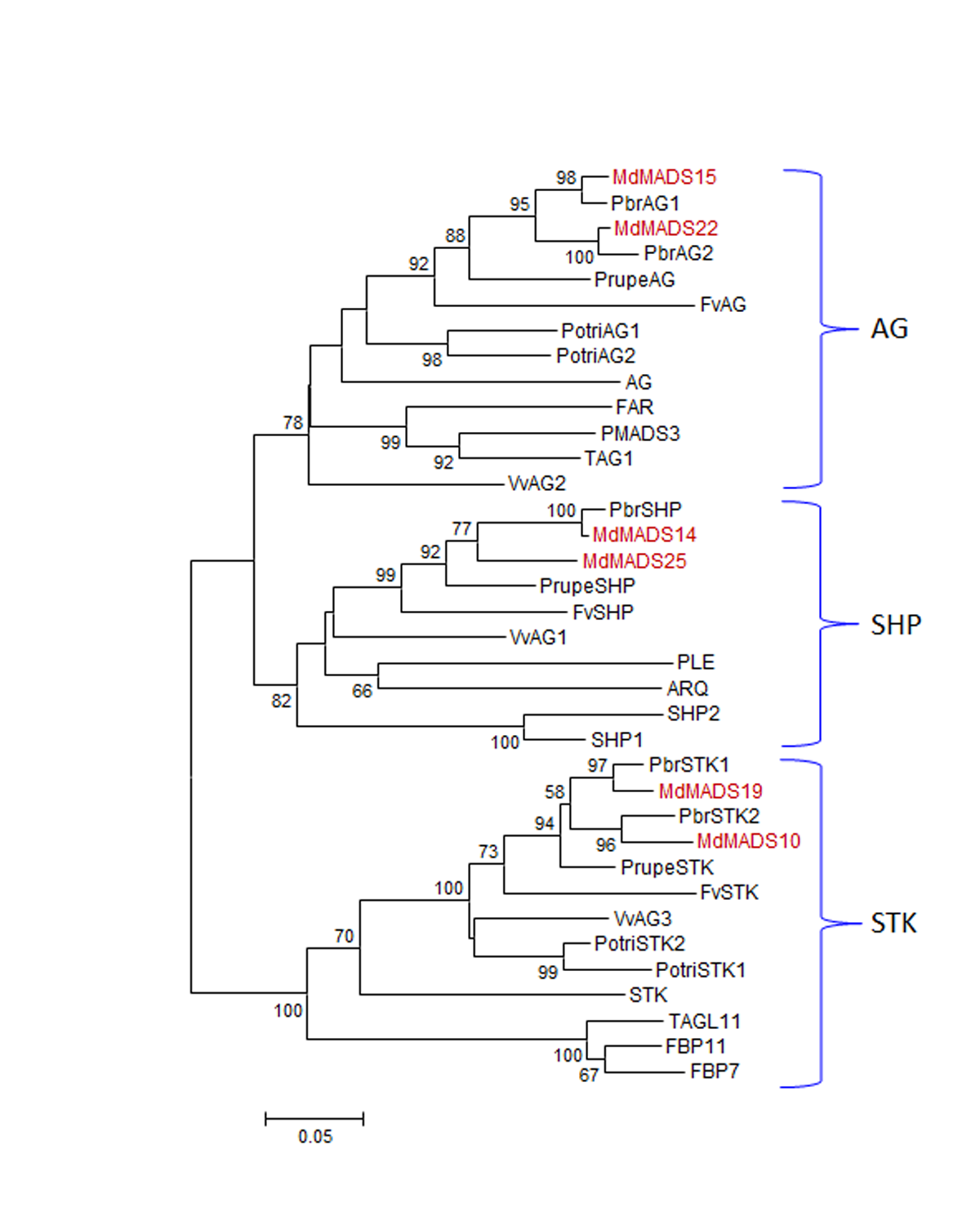

Supplement: S1 Fig — Alignment of protein sequences and the neighbor-joining method were used to produce the tree, and bootstrap values of 50% or higher are indicated at the nodes. Clades are named based on the Arabidopsis thaliana members. All family members identified in the Malus x domestica (prefix Md, shown in red) Prunus persica (Prupe), Pyrus x bretschneideri (Pbr), Fragaria vesca (Fv), Populus trichocarpa (Potri), and Vitis vinifera (Vv) genomes were included. Additional proteins were from Antirrhinum majus (PLE, FAR) Solanum lycoperscium (ALQ, TAG1, TAGL11), Petunia x hybrida (PMADS3, FBP7, FBP11). (TIF) [file pone.0159421.s001.tif]

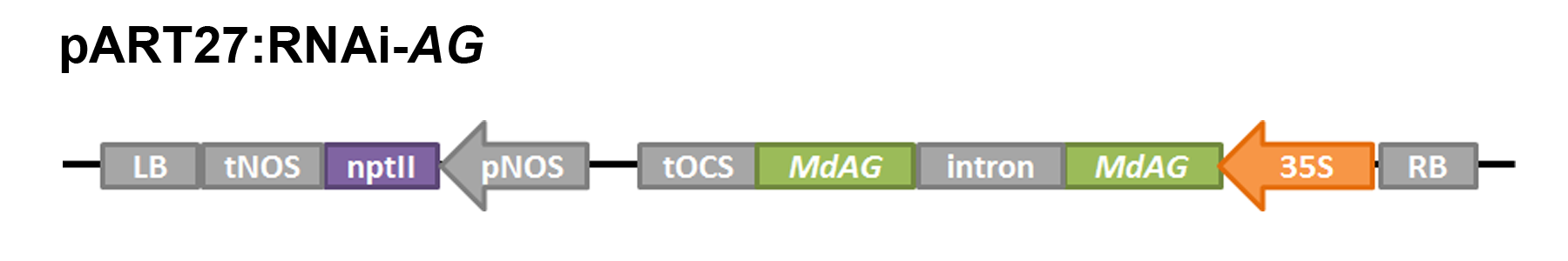

Supplement: S2 Fig — An inverted repeat of a 420 bp fragment of the apple MdMADS22 gene (MdAG) was cloned into the pART27 vector under control of the constitutive 35S promoter and terminated by the octapine synthase 3’ untranslated region (tOCS). This construct contained a neomycin phosphotransferase II (nptII) gene controlled by the constitutive nopaline synthase promoter (pNOS) and terminated by the nopaline synthase 3’ untranslated region (tNOS). Arrows indicate the direction of transcriptional activation. The left border and right border are indicated by LB and RB, respectively. (TIF) [file pone.0159421.s002.tif]

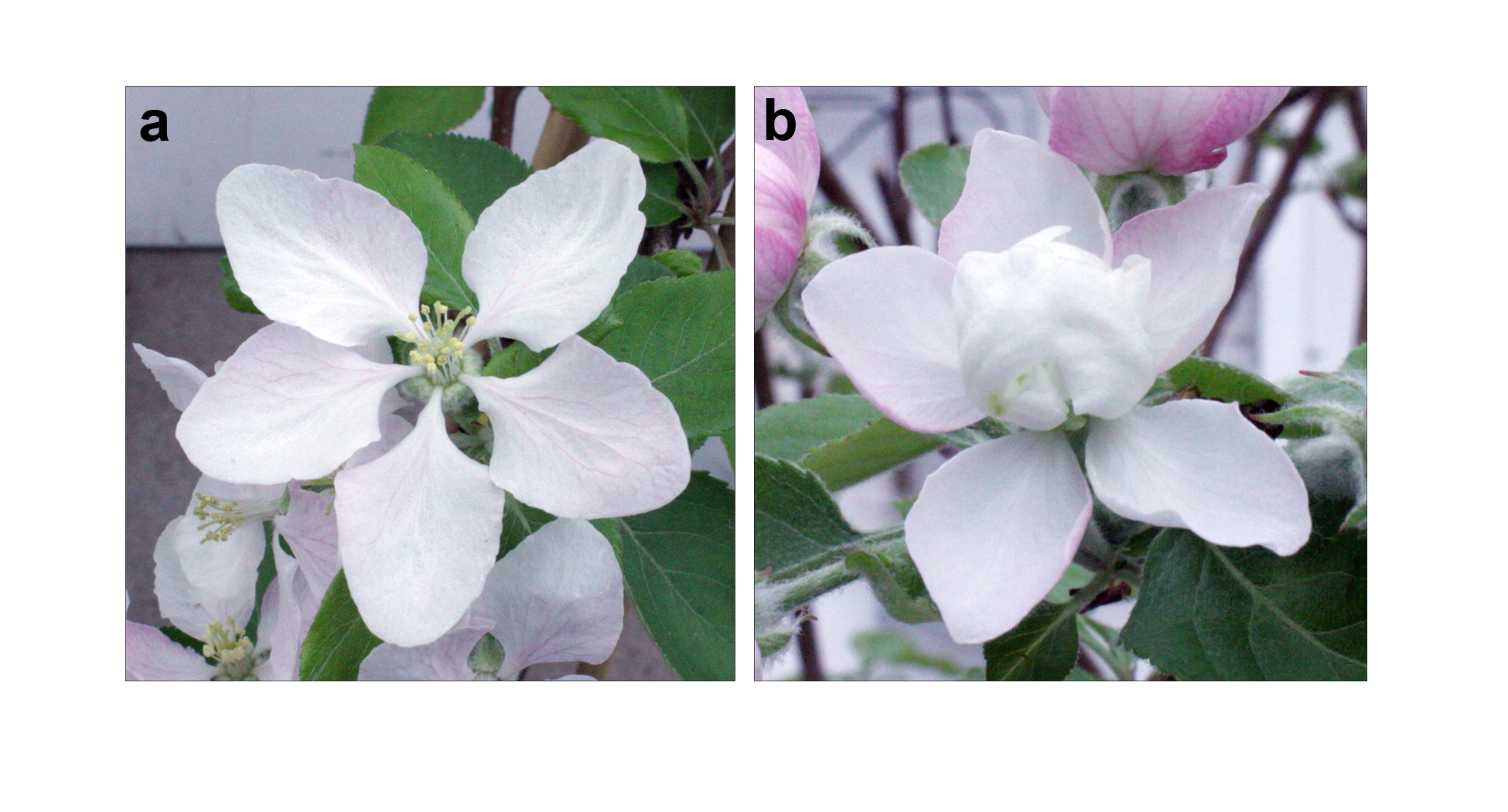

Supplement: S3 Fig — (a) Control trees had five total petals (b) while double-flowers had five outer petals and numerous inner petals. These five outermost petals were highly similar to petals of control trees and opened before the inner petals. (TIF) [file pone.0159421.s003.tif]

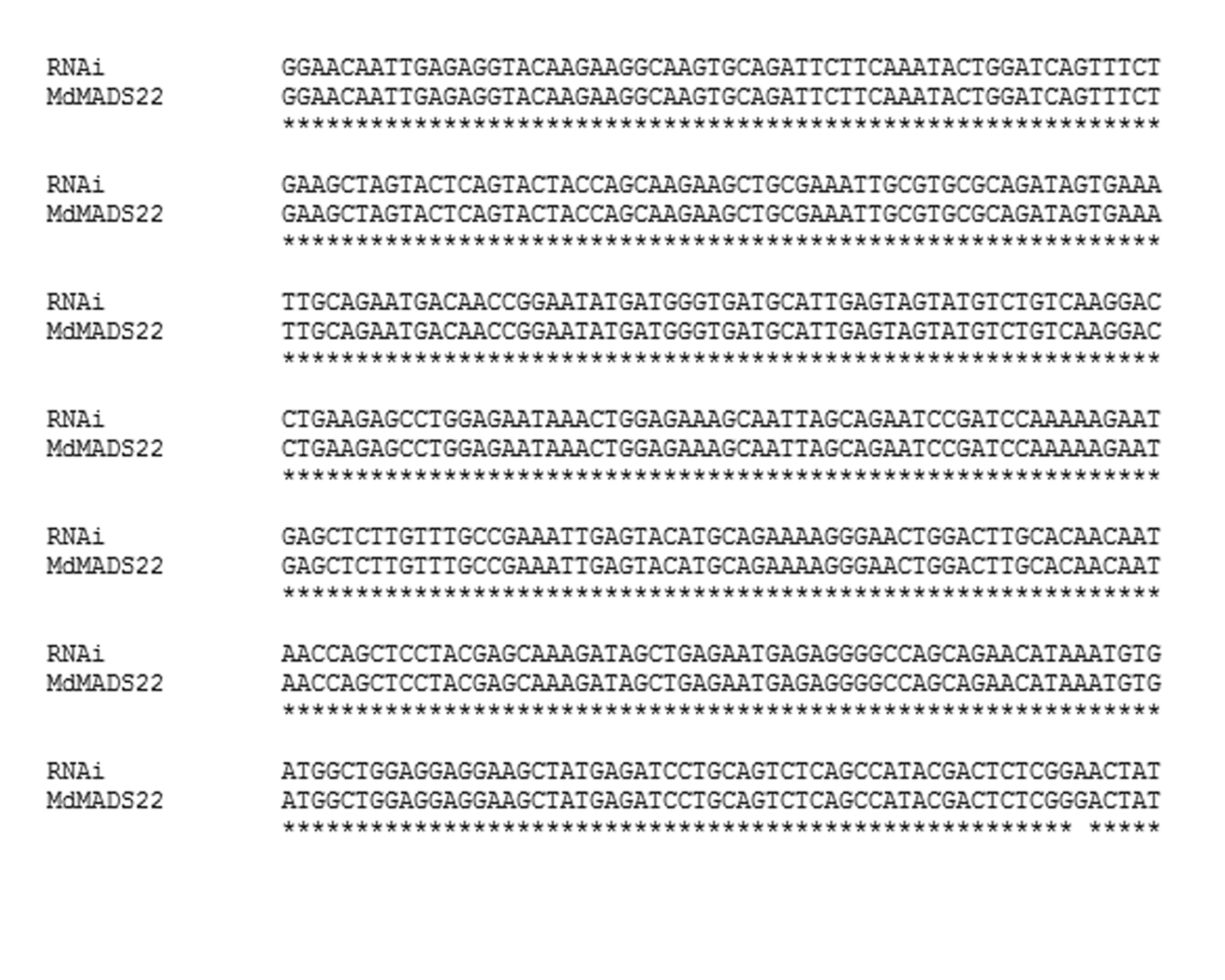

Supplement: S4 Fig — Alignment of the sequence used to target apple AG-like genes with MdMADS22. Perfect matches are indicated by asterisks below the matched bases, dashes indicate gaps, and numbers indicate base pairs. (TIF) [file pone.0159421.s004.tif]

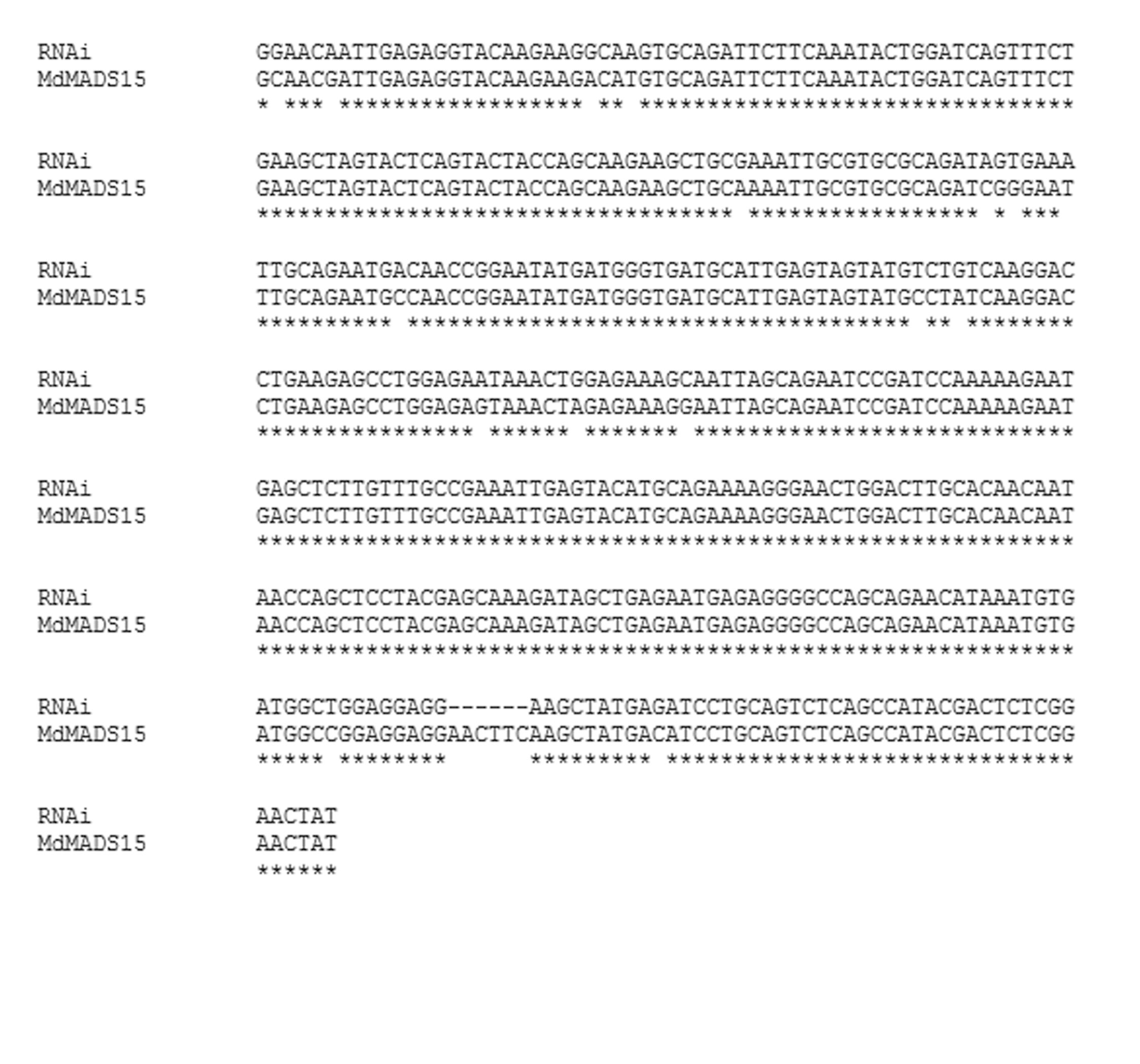

Supplement: S5 Fig — Alignment of the sequence used to target apple AG-like genes with MdMADS15. Perfect matches are indicated by asterisks below the matched bases, dashes indicate gaps, and numbers indicate base pairs. (TIF) [file pone.0159421.s005.tif]

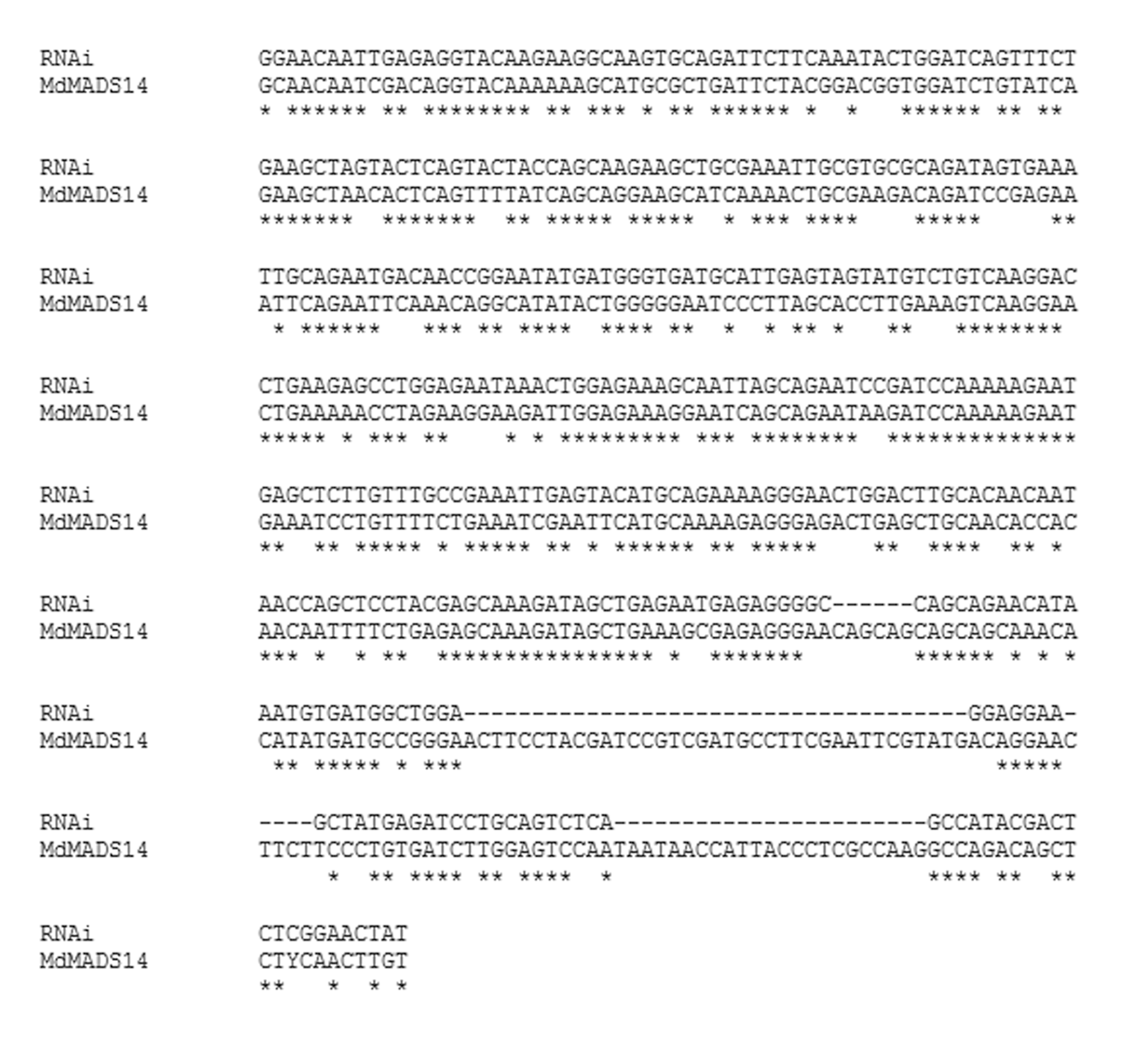

Supplement: S6 Fig — Alignment of the sequence used to target apple AG-like genes with MdMADS14. Perfect matches are indicated by asterisks below the matched bases, dashes indicate gaps, and numbers indicate base pairs. (TIF) [file pone.0159421.s006.tif]

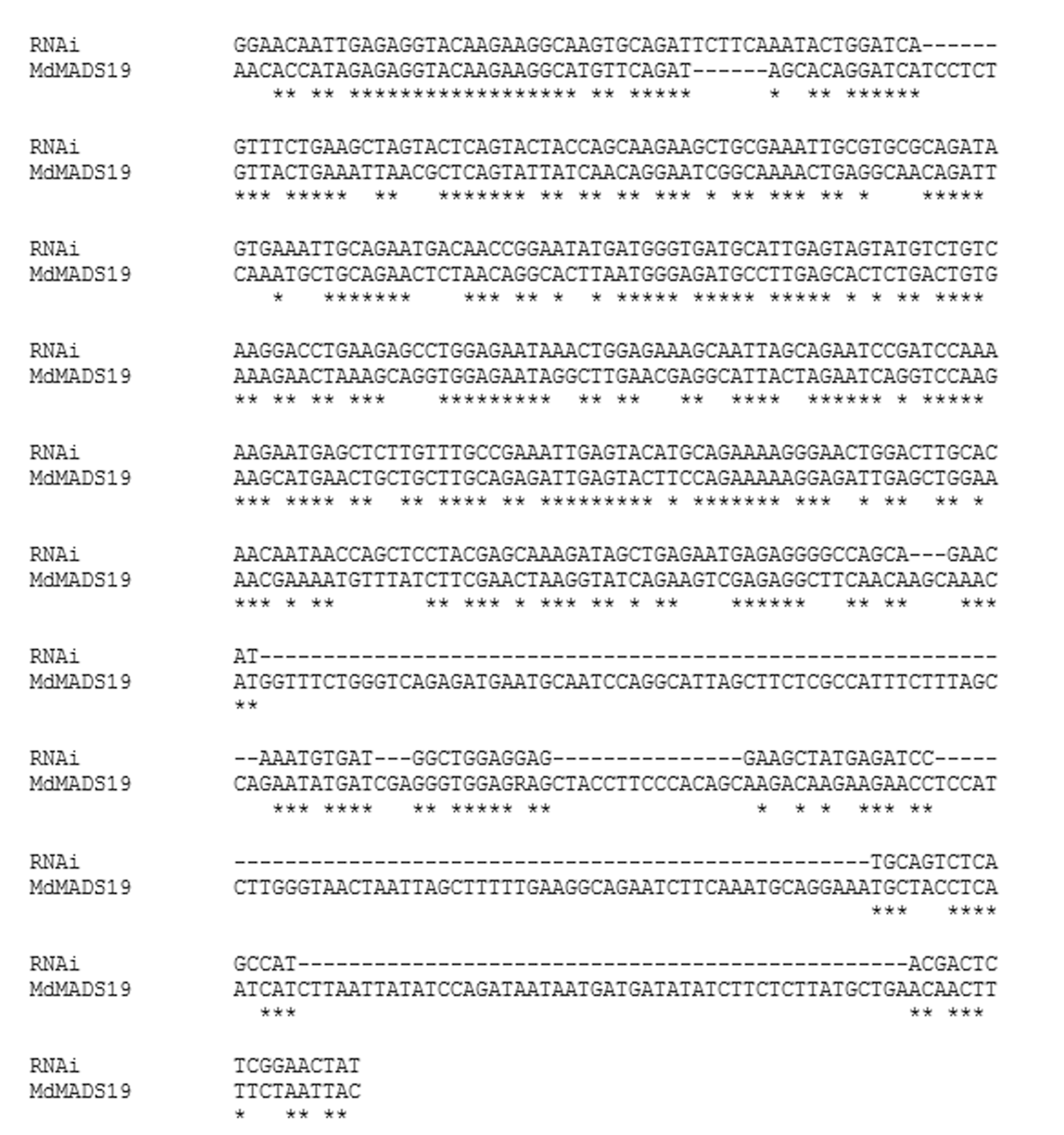

Supplement: S7 Fig — Alignment of the sequence used to target apple AG-like genes with MdMADS19. Perfect matches are indicated by asterisks below the matched bases, dashes indicate gaps, and numbers indicate base pairs. (TIF) [file pone.0159421.s007.tif]

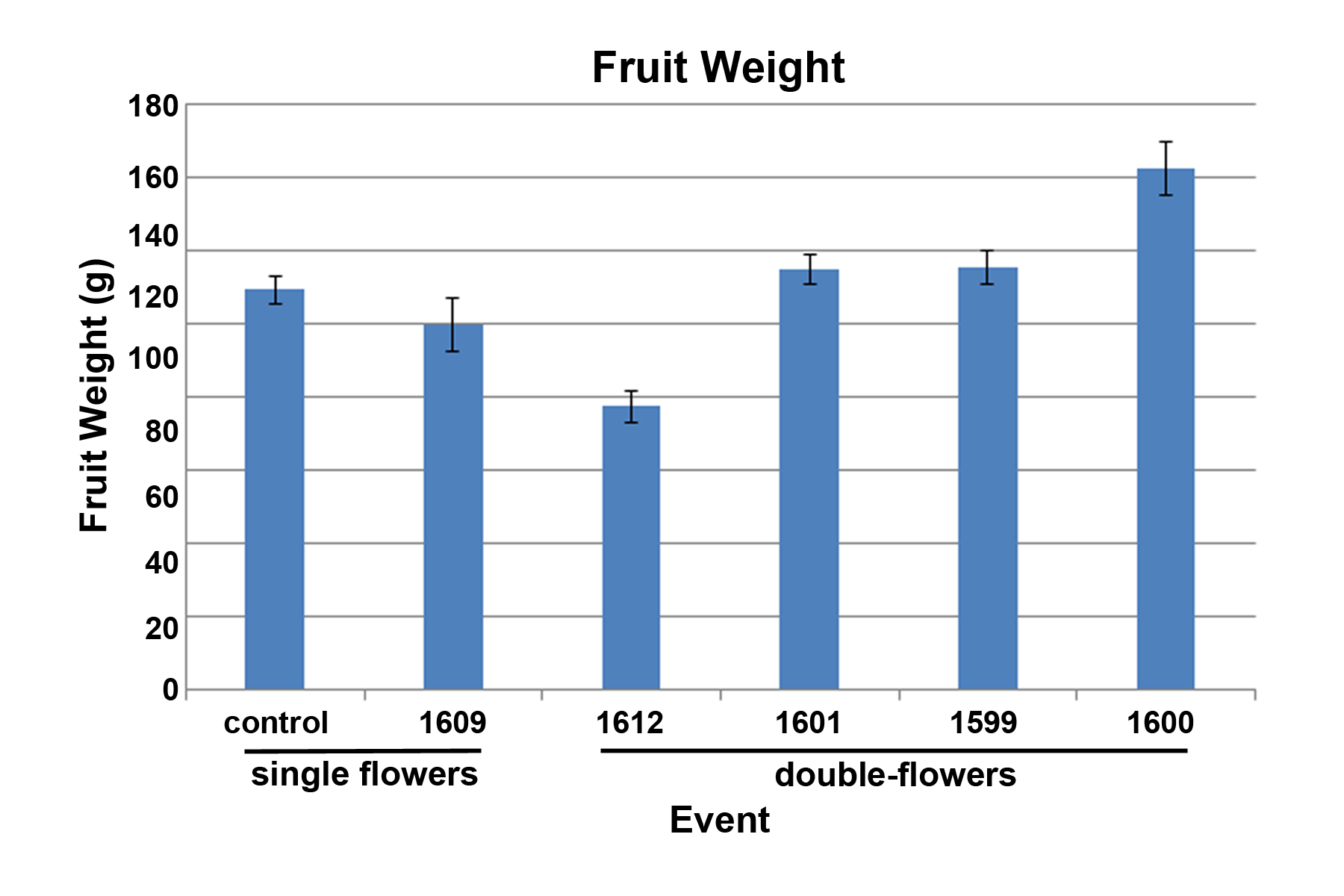

Supplement: S8 Fig — Quantification of fruit size (by weight) showed that fruits produced by RNAi events were as large as fruits produced by control trees. Bars show standard error of the mean. (TIF) [file pone.0159421.s008.tif]

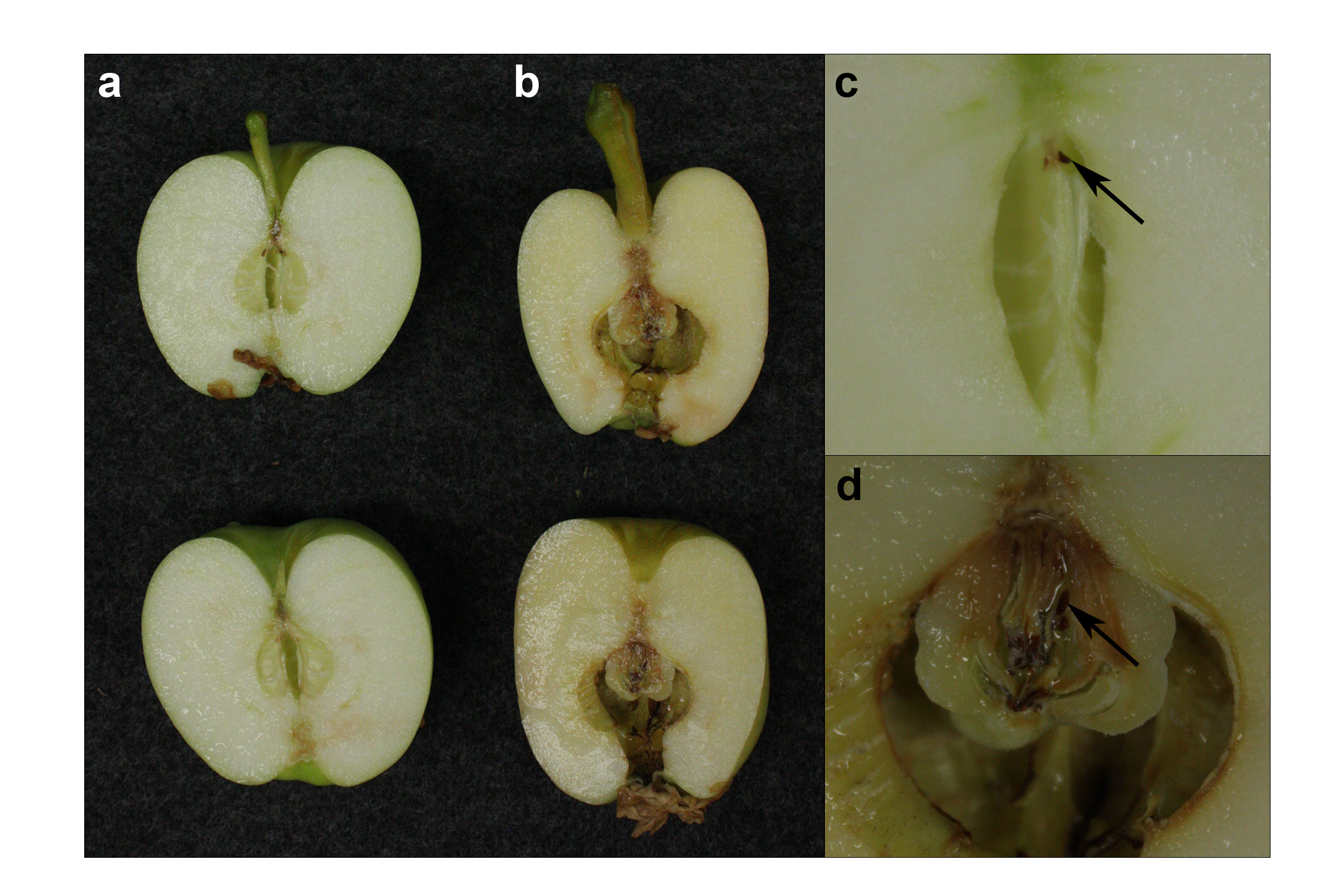

Supplement: S9 Fig — (a) Apples from control trees contained a hollow internal cavity with small undeveloped seeds (c, arrow). (b) Two of the RNAi-AG events set fruit with a large fleshy structure in the center that contained small undeveloped seeds (d, arrow). (TIF) [file pone.0159421.s009.tif]

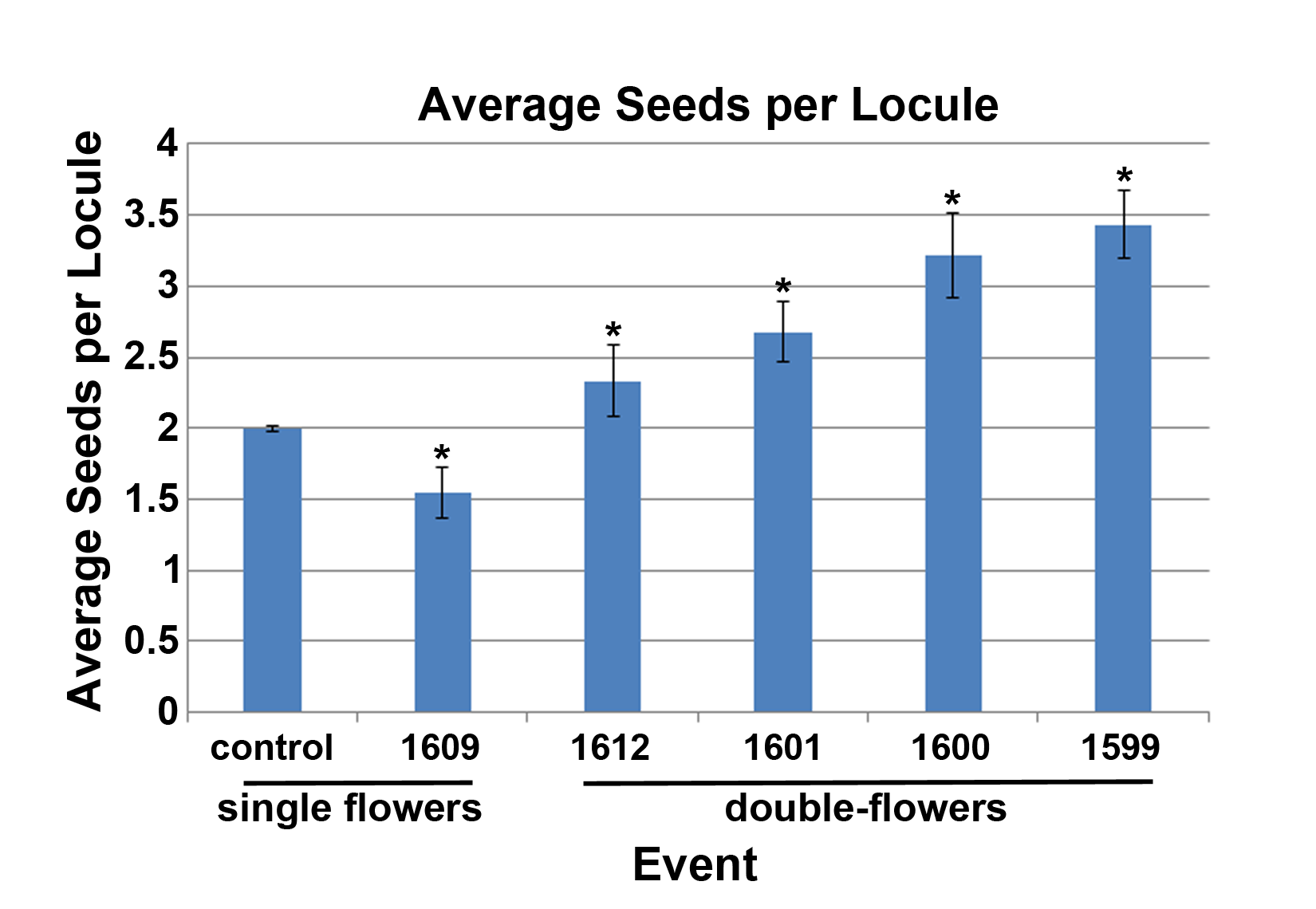

Supplement: S10 Fig — Fruits developed from RNAi-AG events with double-flowers had an increased number of seeds per locule. Bars show standard error, asterisks indicate significant differences (P < .01). (TIF) [file pone.0159421.s010.tif]

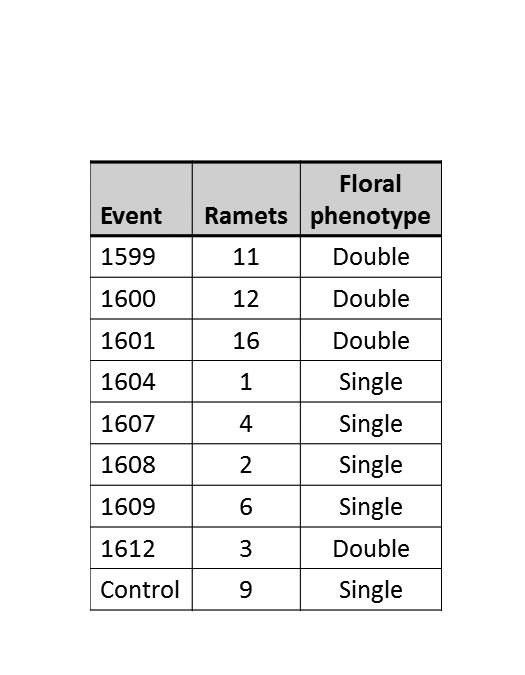

Supplement: S1 Table — Eight independent events represented by 1–16 individual ramets (trees) were tested. Flowers were classified as either single (phenotypically similar to non-transgenic control flowers) or double (possessing extra petals). (TIF) [file pone.0159421.s011.tif]

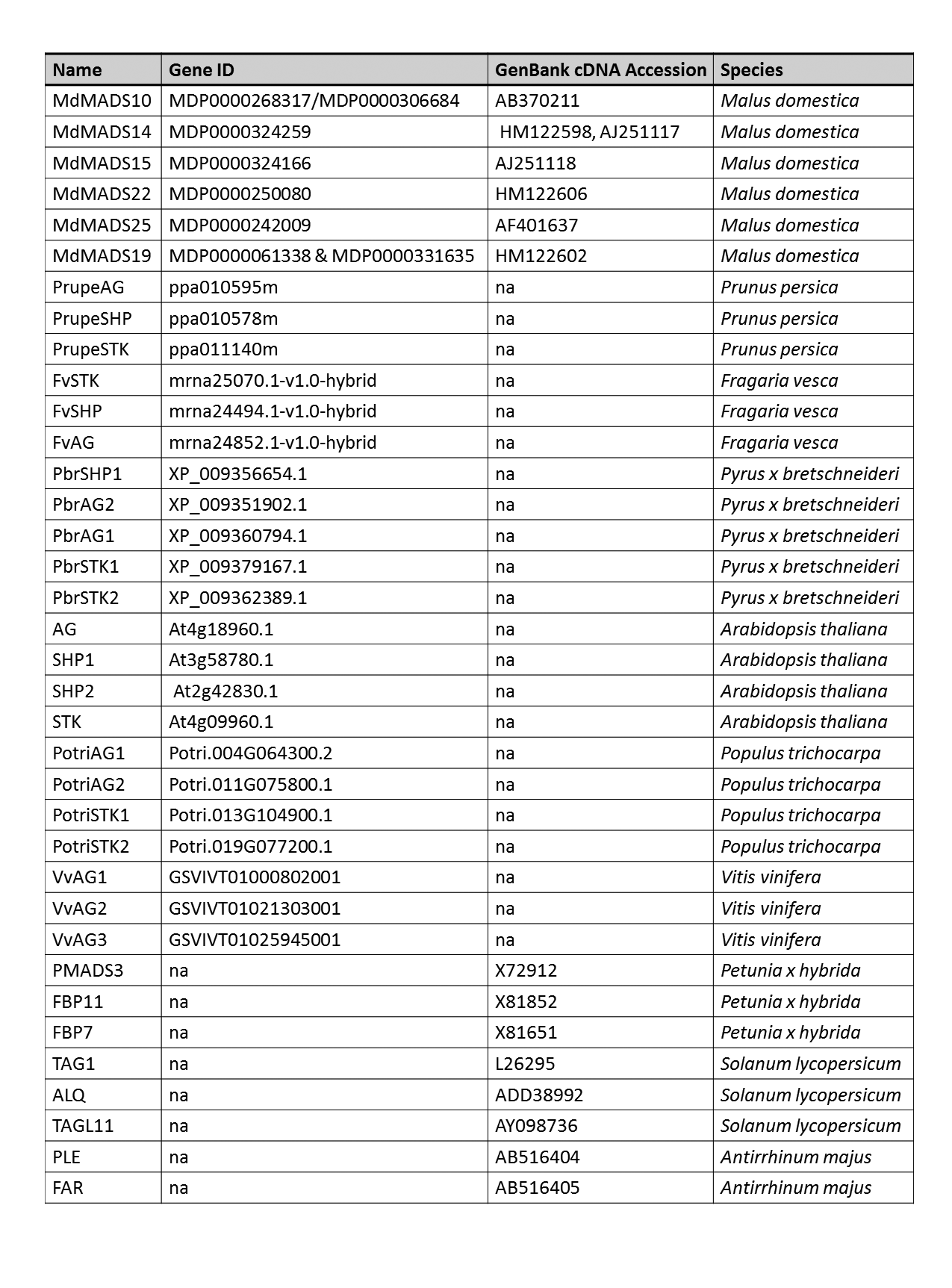

Supplement: S2 Table — The names, gene ID(s), GenBank cDNA accessions, and species for all genes used in the phylogenetic analysis are shown. Three of the MdMADS genes had gene IDs which differed from the GenBank cDNA accessions. Specifically, the MdMADS10 gene ID had two partial gene IDs associated with it. The MDP0000268317 prediction missed calling 3' exons and model MDP0000306884 lacks a MADS-box, likely due to the sequence being located at the end of a scaffold. The cDNA encoded protein was used for phylogenetic analysis. The gene ID for MdMADS25 predicted different (atypical) splicing compared to the cDNA sequence; the cDNA encoded protein was used for phylogenetic analysis. None of the current gene IDs for MdMADS19 matched the GenBank cDNA accession. A combination of alternate models MDP0000061338 and MDP0000331635 resulted in a best fit to the cDNA accession for this gene, na, not applicable. (TIF) [file pone.0159421.s012.tif]
